# Supplementary material for: Development and psychometric properties of the Nursing Student Academic Resilience Inventory (NSARI): A mixed-method study
Source: PLoS One. 2021 Jun 15;16(6):e0252473. doi: 10.1371/journal.pone.0252473 (PMC8205183; doi:10.1371/journal.pone.0252473)
Supplement: Attachment — Submitted filename: Revise PONE-D-20-37513_reviewer.pdf [file pone.0252473.s002.pdf]

PLOS ONE

Development and psychometric properties of the Nursing Student Academic resilience Inventory (NSARI): A Mixed-Method study

--Manuscript Draft--

**Manuscript Number:**	PONE-D-20-37513
**Article Type:**	Research Article
**Full Title:**	Development and psychometric properties of the Nursing Student Academic resilience Inventory (NSARI): A Mixed-Method study
**Short Title:**	Development and psychometric properties of the Nursing Student Academic resilience Inventory
**Corresponding Author:**	ali Asghar ghods<br>Semnan University of Medical Sciences and Health Services<br>semnan, IRAN, ISLAMIC REPUBLIC OF
**Keywords:**	Nursing, student, psychometric, development, Resilience
**Abstract:**	Introduction: Resilience has been proposed as a suitable solution to a better deal with nursing students in the face of challenges and difficulties which lead to better preparation of students to play their professional roles in the future. The complex and multidimensional nature of resilience has made the measurement of this concept to be challenging. This study is fulfilled to develop and validate a theory-driven inventory labeled Nursing Student Academic Resilience Inventory.<br>Methods : This study is an exploratory sequential mixed-method design. In the qualitative phase of the study, individual interviews are conducted with 15 participants to elicit the concept of resilience through purposive sampling. In the quantitative phase, psychometric analysis of the extracted items is performed using face, content, and construct validity (exploratory and confirmatory factor analysis) with a sample size of 405 nursing students. Besides, reliability has been done through internal consistency and test-retest methods. According to the COSMIN standards, in addition to two important indicators of validity and reliability, responsiveness, interpretability also is considered .<br>Results : A 6-factor structure (optimism, communication, self-esteem/evaluation, self-awareness, trustworthiness, self-regulation) with 24 items are extracted based on the derived categories from the qualitative phase. In confirmatory factor analysis, the χ^2/df ratio is 2.11 for the NSARI six-factor structure. Suitable values are obtained for the goodness of fit indices (CFI= 0.904 , AGFI=0.885, IFI= 0.906 , PCFI= 0.767 , RMSEA= 0.053). In the second-order factor analysis, AVE= 0.70 indicated the existence of convergent and divergent validity. The Cronbach's alpha and omega coefficients is investigated to be (0.66- 0.78), and (0.66-0.80), respectively. The AIC is between 0.33 and 0.45 for all factors, which is acceptable. Also an intraclass correlation coefficient (ICC) obtained for the whole instrument is .903 (CI .846- .946, P <0.0001).<br>Conclusion: Multidimensional nature of resilience is supported through exploring its 6-factor structures in the nursing students' field. This tool also shows an acceptable validity and reliability for measuring resilience in the population of nursing students.
**Order of Authors:**	tayyebeh AliAbadi
	Abbas Ebadi
	hamid sharif nia
	mohsen soleimani
	ali Asghar ghods
**Additional Information:**	
**Question**	**Response**
**Financial Disclosure**<br><br>Enter a financial disclosure statement that	The funders had no role in study design, data collection and analysis, decision to publish, or preparation of the manuscript.

Powered by Editorial Manager® and ProduXion Manager® from Aries Systems Corporation

describes the sources of funding for the work included in this submission. Review the submission guidelines for detailed requirements. View published research articles from *PLOS ONE* for specific examples.<br><br>This statement is required for submission and **will appear in the published article** if the submission is accepted. Please make sure it is accurate.<br><br>**Unfunded studies**<br>Enter: *The author(s) received no specific funding for this work.*<br><br>**Funded studies**<br>Enter a statement with the following details:<br>• Initials of the authors who received each award<br>• Grant numbers awarded to each author<br>• The full name of each funder<br>• URL of each funder website<br>• Did the sponsors or funders play any role in the study design, data collection and analysis, decision to publish, or preparation of the manuscript?<br>• **NO** - Include this sentence at the end of your statement: *The funders had no role in study design, data collection and analysis, decision to publish, or preparation of the manuscript.*<br>• **YES** - Specify the role(s) played.<br><br>* typeset	
**Competing Interests**<br><br>Use the instructions below to enter a competing interest statement for this submission. On behalf of all authors, disclose any competing interests that could be perceived to bias this work—acknowledging all financial support and any other relevant financial or non-financial competing interests.<br><br>This statement **will appear in the published article** if the submission is accepted. Please make sure it is accurate. View published research articles from *PLOS ONE* for specific examples.	The authors have declared that no competing interests exist

Powered by Editorial Manager® and ProduXion Manager® from Aries Systems Corporation

**NO authors have competing interests**<br><br>Enter: *The authors have declared that no competing interests exist.*<br><br>**Authors with competing interests**<br><br>Enter competing interest details beginning with this statement:<br><br>*I have read the journal's policy and the authors of this manuscript have the following competing interests: [insert competing interests here]*<br><br>* typeset	
**Ethics Statement**<br><br>Enter an ethics statement for this submission. This statement is required if the study involved:<br><br>• Human participants<br>• Human specimens or tissue<br>• Vertebrate animals or cephalopods<br>• Vertebrate embryos or tissues<br>• Field research<br><br>Write "N/A" if the submission does not require an ethics statement.<br><br>General guidance is provided below. Consult the submission guidelines for detailed instructions. **Make sure that all information entered here is included in the Methods section of the manuscript.**	The present study is the result of a project approved by the Nursing Care Research Center, Semnan University of Medical Sciences with the code of ethics IR.SEMUMS.REC.1398013. In the qualitative phase informed consent obtained was verbal due to in this way participants feel more intimate relationship for participating in interview sessions; and in the quantitative phase data were collected online and they were requested to complete the questionnaire if they consented to participate in the study.

Powered by Editorial Manager® and ProduXion Manager® from Aries Systems Corporation

**Format for specific study types**<br><br>**Human Subject Research (involving human participants and/or tissue)**<br>• Give the name of the institutional review board or ethics committee that approved the study<br>• Include the approval number and/or a statement indicating approval of this research<br>• Indicate the form of consent obtained (written/oral) or the reason that consent was not obtained (e.g. the data were analyzed anonymously)<br><br>**Animal Research (involving vertebrate animals, embryos or tissues)**<br>• Provide the name of the Institutional Animal Care and Use Committee (IACUC) or other relevant ethics board that reviewed the study protocol, and indicate whether they approved this research or granted a formal waiver of ethical approval<br>• Include an approval number if one was obtained<br>• If the study involved *non-human primates*, add *additional details* about animal welfare and steps taken to ameliorate suffering<br>• If anesthesia, euthanasia, or any kind of animal sacrifice is part of the study, include briefly which substances and/or methods were applied<br><br>**Field Research**<br><br>Include the following details if this study involves the collection of plant, animal, or other materials from a natural setting:<br>• Field permit number<br>• Name of the institution or relevant body that granted permission	
**Data Availability**<br><br>Authors are required to make all data underlying the findings described fully available, without restriction, and from the time of publication. PLOS allows rare exceptions to address legal and ethical concerns. See the PLOS Data Policy and FAQ for detailed information.	Yes - all data are fully available without restriction

A Data Availability Statement describing where the data can be found is required at submission. Your answers to this question constitute the Data Availability Statement and **will be published in the article**, if accepted.<br><br>**Important:** Stating 'data available on request from the author' is not sufficient. If your data are only available upon request, select 'No' for the first question and explain your exceptional situation in the text box.<br><br>Do the authors confirm that all data underlying the findings described in their manuscript are fully available without restriction?	
**Describe where the data may be found in full sentences. If you are copying our sample text, replace any instances of XXX with the appropriate details.**<br><br>• If the data are **held or will be held in a public repository**, include URLs, accession numbers or DOIs. If this information will only be available after acceptance, indicate this by ticking the box below. For example: *All XXX files are available from the XXX database (accession number(s) XXX, XXX.)*.<br>• If the data are all contained **within the manuscript and/or Supporting Information files**, enter the following: *All relevant data are within the manuscript and its Supporting Information files.*<br>• If neither of these applies but you are able to provide **details of access elsewhere**, with or without limitations, please do so. For example:<br><br>*Data cannot be shared publicly because of [XXX]. Data are available from the XXX Institutional Data Access / Ethics Committee (contact via XXX) for researchers who meet the criteria for access to confidential data.*<br><br>*The data underlying the results presented in the study are available from (include the name of the third party*	All relevant data are within the manuscript and its Supporting Information files.

and contact information or URL).<br>• This text is appropriate if the data are owned by a third party and authors do not have permission to share the data.<br><br>* typeset	
Additional data availability information:	Tick here if your circumstances are not covered by the questions above and you need the journal's help to make your data available.

Manuscript Click here to access/download;Manuscript;manuscript.docx

Development and psychometric properties of the Nursing Student Academic resilience Inventory (NSARI): A Mixed-Method study

Tayyebeh Ali-Abadi[1], Abbas Ebadi[2], Hamid Sharif Nia [3], Mohsen Soleimani[4], Ali-Asghar Ghods[5]*

1-Student Research Committee, Semnan University of Medical Sciences, Semnan, Iran. Mobile: +98153529801. Email:tayebealiabadi@yahoo.com

2- Ph.D., Professor in Nursing Education Behavioral Sciences Research Center, Lifestyle Institute, Nursing Faculty, Baqiyatallah University of Medical Sciences, Tehran, IR Iran. Mobile: +98 912 214 90 19, Fax: +98 21 82483443 P.O.Box:19575-174. E-Mails:ebadi1347@yahoo.com & ebadi1347@bmsu.ac.ir

3- Hamid Sharif Nia, BScN, MScN, Ph.D. in Nursing Education, Assistant Professor Department of Nursing, Mazandaran University of Medical Science, Sari, Iran. Tel: +981144221919. Mobile: +989111275093. Email: sharifnia@mazums.ac.ir, pegadis@yahoo.com

4- Nursing Care Research Center, Semnan University of Medical Sciences, Semnan, Iran. Mobile: +98122977445 . Email: soli257@yahoo.com.

5- Nursing Care Research Center, Semnan University of Medical Sciences, Semnan, Iran. Mobile: +98122319532 . Email: aaghods@yahoo.com ,***Corresponding author**

Development and psychometric properties of the Nursing Student Academic resilience Inventory (NSARI): A Mixed-Method study

Abstract

Introduction: Resilience has been proposed as a suitable solution to a better deal with nursing students in the face of challenges and difficulties which lead to better preparation of students to play their professional roles in the future. The complex and multidimensional nature of resilience has made the measurement of this concept to be challenging. This study is fulfilled to develop and validate a theory-driven inventory labeled Nursing Student Academic Resilience Inventory.

Methods: This study is an exploratory sequential mixed-method design. In the qualitative phase of the study, individual interviews are conducted with 15 participants to elicit the concept of resilience through purposive sampling. In the quantitative phase, psychometric analysis of the extracted items is performed using face, content, and construct validity (exploratory and confirmatory factor analysis) with a sample size of 405 nursing students. Besides, reliability has been done through internal consistency and test-retest methods. According to the COSMIN standards, in addition to two important indicators of validity and reliability, responsiveness, interpretability also is considered.

Results: A 6-factor structure (optimism, communication, self-esteem/evaluation, self- awareness, trustworthiness, self-regulation) with 24 items are extracted based on the derived categories from the qualitative phase. In confirmatory factor analysis, the χ^2/df ratio is 2.11 for the NSARI six-factor structure. Suitable values are obtained for the goodness of fit indices (CFI=0.904, AGFI=0.885, IFI=0.906, PCFI=0.767, RMSEA= 0.053). In the second-order factor analysis, AVE= 0.70 indicated the existence of convergent and divergent validity. The Cronbach's alpha and omega coefficients is investigated to be (0.66- 0.78), and (0.66-0.80), respectively. The AIC is between 0.33 and 0.45 for all factors, which is acceptable. Also an intraclass correlation coefficient (ICC) obtained for the whole instrument is .903 (CI .846- .946, P <0.0001).

Conclusion: Multidimensional nature of resilience is supported through exploring its 6-factor structures in the nursing students' field. This tool also shows an acceptable validity and reliability for measuring resilience in the population of nursing students.

Key words: Nursing, student, psychometric, development, Resilience

Introduction

Research conducted in Iran showed that nursing students are affected by various stressors such as lack of effective communication between trainer and students, unclear training goals, lack of essential efficacy when attending the patient's bedside, fear of infectious diseases, assignments overload, and dissatisfaction with the field of the study [1]. The issue of stress in nursing students requires further study; due to the detrimental effects of high stress including poor commitment to clinical internships, lower self-esteem, and lack of physiological and mental health, besides Low-quality patient care, as well as students` drop out [2].

Resilience has been suggested as a beneficial solution for better dealing with challenges and difficulties in nursing students, which leads to excel for playing their professional roles in the future [3]. It is documented that students with higher academic resilience maintain a foremost level of academic achievement and performance even in the stressful or failure situation [4]. A systematic review study by Ray et al. (2015) shows that nursing students and trainers face prime challenges that necessitate resilience [5] therefore, academic resilience is essential for nursing students [4].

Resilience is described as a characteristic, process or outcome hinges on the theory accepted by the researcher [6]. Theories can be effective in identifying and understanding the factors influencing an event or behavior and determining how they work together; therefore, a conceptual model can lead to a better understanding of the existing conditions [7]. In line with the different resilience theories in this study Stephen's resilience model is used which emphasizes the importance of defining the concept of resilience as a process. She declares “as nursing students learn to identify, enhance, and/or develop their protective factors, they will be better equipped to effectively manage perceived adversity and stress. The cumulative successes of

these events will lead to increased resilience demonstrated by enhanced coping/adaptive abilities and well-being” [3].

Diverse approaches to measuring resilience affected by different nature of potential risk factors, and protective processes have led to contradictions [8]. While establishing the resilience concept as a meaningful concept in research and clinical practice, it is inevitable to determine its distinctive factors and measure its factors in a valid and reliable method [9]. Based on the result of a review study yielded by Chmitorz et al. (2018) various resilience scales have been developed [10]. Different studies have founded different factor structures of resilience tools in different populations and cultures [11, 12].

As a result, researchers and clinicians do not have strong evidences about the choice of resilience measurement tools and may make inappropriate choices in the field of study [13]. Notwithstanding the prevalence of stress and the obligation to design an appropriate tool to assess students' resilience, it is not yet clear which factors measure the resilience in nursing students. The purpose of this study is to develop and design a valid and reliable inventory for measuring resilience in nursing students through an exploratory sequential mixed-methods design.

Methods

This exploratory sequential mixed-method study is conducted between May 2019 and August 2020 in Iran. The study population includes all bachelors nursing students. The data in the qualitative phase is collected through semi-structured interviews based on resilience Stephen's model in nursing students. Item generation and developing inventory are done based on the

categories extracted from the qualitative phase. Then, psychometric properties are evaluated in the quantitative phase.

Qualitative phase

Data collection

In this phase, semi-structured individual interviews are conducted with thirteen nursing students aged 18- 25 years, one nurse, and one trainer using purposive sampling in a quiet room. Exploratory questions are asked following the main questions that are derived from the Stephen resilience model. The main questions include “explain about the situation experienced stress during nursing education.”, “which protective factors did you apply in these situations?”, and “explain about cumulative success do you have acquired?”. To achieve maximum data diversity students are selected of both gender, different semesters, private and public universities. The interviews are perpetuated until data saturation.

Data analysis

The method of analysis is the deductive content analysis approach which is recommended by Elo et al. [14]. The interviews are audio-recorded, transcribed, and consequently analyzed using MAXQDA software version 10. Data analysis is performed during three stages of preparation, organization, and reporting. During the preparation phase, semantic units are identified based on the purpose of the study. To get immersed in the data, the written interviews are read through several times. To make sense, the researcher frequently asks wh-questions during the analysis. In the organizing phase the text is read line by line, and paragraph by paragraph according to the purpose of the study, hence important sentences and codes are identified. After this open coding, the lists of categories are classified under higher-order headings based on the comparison between these data and other observations that do not associate with the exact category. To

assure data credibility, samples are selected with maximum variation, and also peer checking is done by three co-authors experienced in qualitative studies [14]. Hence, the first draft of the inventory is developed based on the findings of the directed content analysis.

Quantitative phase

In the quantitative phase, the psychometric properties of the Nursing Student Academic Resilience Inventory are assessed using face, content, and construct validity (exploratory factor analysis, confirmatory).

Face and Content validity

Following revising items of the designed inventory according to the comments of 13 nursing students, in the qualitative face validity stage, quantitative face validity is assessed using the impact factor method, based on the following equation:

Impact Score = Frequency × Importance

The items which equal and above 1.5 are retained and other items are modified [15]. Content validity ratio (CVR) determined essential items according to the cut-off point proposed by Lawshe [16]. The Content Validity Index for each item (ICVI), and then the modified kappa coefficient is calculated based on the following equation[17]. Finally, S-CVI is estimated.

$$K = \frac{\text{I.CVI} - \text{PC}}{1 - \text{PC}} \rightarrow \text{PC} = \left[\frac{N!}{A!(N-A)!} \right] \times 0.5^{N}$$

Item Analysis

The internal consistency is assessed in the population of 36 nursing students before construct validity assessment in a pilot study to recognize potential problems in the NSARI through

estimating Cronbach's alpha and inter‑ item correlation. The items whose corrected item-total correlation score is less than 0.3 are removed from the analysis [18].

Normal distribution, Outliers, and Missing data

The univariate and multivariate normal distribution of data are investigated by skewness (±3) and kurtosis (±7) [19]. The presence of a multivariate outlier is assessed by Mahalanobis d-squared ($p < .001$), and multivariate normality by Mardia coefficient of multivariate kurtosis (< 20), respectively [20]. There is no missing data in the study due to completing the surveys by an online Survey software (Porsline). All the statistical analyses are calculated by SPSS- $AMOS_{25}$ and SPSS R-Menu v2.0.

Construct Validity

Exploratory Factor Analysis

Exploratory factor analysis is conducted to test the factor structure of the items of the NSARI so in a cross‑ sectional study 200 nursing students of private and public universities from 6 different provinces in Iran participated in the study through convenience sampling. The Kaiser-Meyer-Olkin statistic of sampling adequacy and the Bartlett test of sphericity are calculated to check the suitability of the data for factor analysis. KMO 0.8 is considered acceptable. In hence, the latent factors are extracted employing the maximum likelihood estimation, and an oblique factor rotation technique, promax, The allocation of an item to a factor is determined based on the formula $CV = 5.152 \quad \sqrt{} (n -2)$ which is approximately 0.3 [21]. According to the three indicator rule, at least 3 items must be retained for each factor in the EFA [22]. Items with communalities less than 0.2 are removed from the EFA [23].

Confirmatory Factor Analysis

Confirmatory factor analysis has been done on a different sample including 205 nursing students to examine fit indices of the extracted factors using Amos 24 software. Table 2 shows the accepted fit indices (CMIN / DF, RMSEA, PCFI, IFI, CFI, PNFI) [19]. In the second-order factor analysis, it is supposed that the extracted latent factors in the first-order factor analysis are a reflection of more general concept in the upper levels [24], therefore the second-order factor analysis is performed following the first-order factor analysis.

Convergent and Divergent validity

Convergent and divergent validity is evaluated based on the average variance extracted (AVE), the maximum variance (MSV). The convergent validity is generated when AVE> 0.5 and divergent validity is confirmed when MSV <AVE [25].

Reliability, Responsiveness, and Interpretability

Internal consistency of the NSARI is estimated using coefficients of Cronbach’s alpha, and Omega McDonald [26]. Values above 0.6 are considered acceptable[27, 28]. AIC above 0.2- 0.4 is acceptable [29]. Construct reliability, which replaces Cronbach's alpha in the structural equation model, is considered as acceptable in the values above 0.7[30]. Test-retest reliability is conducted to investigate the questionnaire’ stability. 36 nursing students were requested to fill the questionnaire twice with a two-week interval. In this study, responsiveness is determined also by the standard error of measurement (SEM) and the minimal detectable change (MDC) score [31]. The MDC less than 30% is acceptable, and below 10% is considered excellent. According to the COSMIN standards, interpretability, and scoring similarly are considered as important capabilities of a tool, in addition to validity and reliability [32]. To determine the interpretability of the NSARI, the distribution of total scores in the whole samples, floor, and ceiling effects are

calculated. It is considered that the floor and ceiling effects are present if more than 20% of respondents attained the lowest or the highest score, respectively [33].

Trustworthiness in the mixed-method study

To diminish the threats to the internal and external validity of the mix-method study, participants are selected with different experiences; also none of the samples in the qualitative phase participate in the quantitative phase. Furthermore, designing the item poll is based on the main categories and sub-categories which are extracted in the qualitative phase. Consequently, all stages of the study are carefully reviewed and verified by other researchers [34].

Ethical consideration

This study is approved by the ethics committee of Semnan University of Medical Sciences (Approval Number IR.SEMUMS.REC.1398013). The volunteering and confidentiality of data are explained to the participants.

Results

Qualitative phase

After condensing the codes extracted from the semantic units, 797 codes are gained. These codes are allocated to three themes, 9 categories, and 31 subcategories. Moreover, an item pool consisting of 93 items are generated that explain the resilience of nursing students based on the directed content analysis. The number of items is reduced to 83 items, following discussion with the research team.

Quantitative phase

In the psychometric evaluation phase, confusing items are revised and duplicate, redundant items are merged and essential items are added based on the results of the face and content validity. The clarity of the items in the initial item pool is evaluated by 13 nursing students through quantitative face validity and then the results of calculating the impact score of each item shows that all of the 83 items extracted are suitable for measuring resilience. According to the results of the content validity ratio (CVR), items with a numerical value of less than 0.59 (11 experts) are removed, and 52 items remain. Finally, after removing the items with the kappa index less than 0.74, 45 items are sent to the nursing students for item analysis. SCVI inventory is 0.92.

Item Analysis

Cronbach's alpha for 45 NSARI items is 0.88. Intra-class correlation coefficient of NSARI total score specifies a high level of temporal stability (*ICC* = 0.88, *95% CI:* 0.82-0.93). Finally, 13 items are eliminated from the analysis.

Construct Validity

The mean age of the participants is 21.67 ±2.72 years. The majority of the students are girls (120). The adequacy index of sampling is 0.892. The Bartlett's test is statistically significant (df= 325, $\chi 2 = 3123.231$ ، $p < 0.001$). During exploratory factor analysis, 8 items are removed due to their low factor loading. The remaining 24 items are loaded on six factors which explain 45.47% of the total variance. Factors that are extracted are labeled based on their items, and their similarity with the dimensions of NSARI is discovered in the qualitative phase of the study (Table 1).

Table1. Exploratory factor analysis of the NSARI

Factor	Items	Factor loading	h^2 [a]	Eigenvalue	Variance (%)
**optimism**	**Q12.** There are many opportunities in	.789	0.58	2.82	11.75

	nursing field for me				
	**Q20**. I am looking forward to be a great nurse in near future.	.739	0.58		
	**Q21.** I am looking forward to an economic prosperity in the field of nursing.	.719	0.37		
	**Q17.** I have a positive attitude toward nursing.	.712	0.56		
	**Q29.** I am motivated to participate in internships.	.360	0.36		
**communication**	**Q24.** Patient companion reduces my stress.	.739	0.47	1.97	8.2
	**Q26.** Understanding my terms by my instructor, reduces my stress	.641	0.49		
	**Q25.** Cooperation by nurses reduces my stress.	.584	0.38		
	**Q23.** Having a good communication with attending physicians reduces my stress.	.420	0.40		
**Self-esteem/ evaluation**	**Q5.** My instructors trust my judgment in taking care of the patients.	.706	0.56	1.53	6.4
	**Q4.** I get support from my instructors.	.643	0.38		
	**Q10.** I have sufficient confidence in taking care of patients.	.344	0.44		
	**Q15.** I have adequate knowledge in (science of) nursing.	.316	0.29		
**Self-awareness**	**Q9.** I manage the difficulties of my academic years.	.825	0.62	1.58	6.6
	**Q11.** I'm not disappointed by the failures during my education.	.521	0.45		
	**Q30.** I have adequate motivation to participate in theory sessions.	.365	0.36		
**trustworthiness**	**Q2.** I earn my patient's trust by making a suitable communication.	.570	0.48	1.41	5.9
	**Q1.** By strengthening my nursing knowledge, I will do my best to take care of the patient.	.470	0.50		

	**Q6.** I get support from my family.	.439	0.34		
	**Q7.** My friends and colleagues support me.	.420	.31		
**Self-regulation**	**Q14.** I examine all options to reach my goals.	.783	0.43	1.59	6.62
	**Q22**. My attempts are to strive and reach my goals.	.461	0.41		
	**Q32.** I learn better by executing bedside procedures.	.393	.36		
	**Q13.** I try to endure the academic hardship.	.336	.37		

a. Communalities

Confirmatory factor analysis

The results of the Chi-square test and other fitness indices confirm the suitability of the studied pattern in the first and second-order factor analysis (Table 2).

Table2. Fitness indices in the first and second-order factor analysis

Indexes	Cut-off values	First-order	Second-order
**CMIN/DF**	< 3	2.11	2.23
***P*- value**	≥ 0.05	<.0001	<.0001
**RMSEA**	≤ 0.08	0.053	0.055
**PCFI**	≥ 0.5	0.767	0.783
**[illegible]I**	≥ 0.95	0.904	0.890
**IFI**	≥ 0.90	0.906	0.891
**AGFI**	≥ 0.90	0.885	0.875
**PNFI**	≥ 0.5	0.708	0.721

According to the final factor structure of the NSARI, correlations between the measurement errors of items4 and 5, 11 and 30, 6 and 7are detected (Fig. 1). The second-order factor analysis is carried out to investigate whether all the factors fit the general concept of “Resilience” or not. Table 2 presents the indices of fit for the first-order CFA compared to the second-order model. Figure 2 shows the structural model and the CFA of the NSARI.

Convergent and divergent validity, internal consistency

The convergent and divergent validity of the NSARI is not confirmed in the first-order factor analysis due to the presence of a latent factor which indicates that all extracted factors are a reflection of concept called "Resilience". The results of the second-order factor analysis (AVE = 0.70) indicate the existence of convergent and divergent validity. The internal construct reliability is confirmed using Cronbach's alpha coefficients and McDonald omega. The AIC is between 0.33 and 0.45 for all factors, which is acceptable (Table 3). The ICC is calculated through the test-retest method which is 0.903(CI .846- .946, P <0.0001) for the whole inventory.

Table3. Internal consistency and construct reliability of NSARI

Factor	α	AIC	Ω	CR*
**optimism**	0.787	0.450	0.806	0.80
**communication**	0.678	0.367	0.701	0.70
**Self-esteem/evaluation**	0.685	0.357	0.691	0.65
**Self-awareness**	0.631	0.371	0.664	0.70
**trustworthiness**	0.647	0.342	0.675	0.65
**Self-regulation**	0.662	0.330	0666	0.66

***CR: Construct Reliability**

Responsiveness and Interpretability

The minimum detectable change percentage is calculated below ten percent (8.61%) and the sstandard error of measurement is determined (3.12). The effect of ceiling and floor on the total inventory scores are estimated less than 20%. The mean and standard deviation of resilience score is different in students with different gender, high and low GPA, semester and different ages. The results of t-test and ANOVA show that all variables except gender and age variables have a significant difference between resilience scores (Table 4).

Table4. Distribution of resilience scores in nursing students

Variable		Mean± SD	Result
**semester**	1	116.88±31	F=3.29; df= 7; p=0.002
	2	128.31±16.27	
	3	122.48±17.29	
	4	122.52±14.44	
	5	130.28±11.53	
	6	120.25±16.16	
	7	133.55±14.73	
	8	124.79±16.87	
**GPA**	<17	123.32±16.92	t=-3.10; df= 382; p=0.002
	>17	130±14.56	

Scoring Items

Each item is rated on a five-point likert scale (“completely agree”, “agree”, “no idea”, “disagree”, and “completely disagree”). The scores of the inventory are recoded to zero to one hundred; then the following scores are transformed to standard scores through the linear scoring using the below formula:

$$\textit{Transformed scale} = \left(\frac{\text{Actual row score} - \text{lowest possible raw score}}{\text{Possible raw score range}} \right) * 100$$

Obviously, after converting the scores to the standard values, the higher average score of resilience close to one hundred means the higher score of the resilience in nursing students.

Discussion

This is the first study with an exploratory sequential mixed-method design to develop an inventory for measuring the resilience in nursing students. Numerous tools have been designed in different contexts, indeed resilience is a context-based concept [35]. In this study, the NSARI

including 24 items, based on extracted categories in the qualitative phase, is designed. The psychometric of the NSARI shows the validity and reliability of the inventory. The internal consistency of the inventory detects that all items measure resilience. Regarding reproducibility, the result of ICC identifies that this inventory can probably catch up with reliable results at different times and places. The Cronbach's alpha coefficient is obtained in the range of 0.63-0.78. Nunnally et al. (1994) [28], and Moss (1998)[27] reported 0.6 as an acceptable value [28]. Based on exploratory factor analysis, six-factors including optimism, communication, self-esteem/evaluation, self-awareness, trustworthiness, and self-regulation are extracted which explain 45.47% of the total variance, similarly review literature show the existence of instability in the factor structure of the resilience scales in different populations [36]. The first-factor extracted in the present study is optimism. Optimism is people's positive expectations of what is happening in their lives [37]. A positive optimistic attitude can help to deal with stress more effectively [38]. Numerous studies have shown the relationship between optimism and students' ability to adapt as well as their educational performance so that Soares et al. (2011) declare that a low level of optimism is a predictor of problems such as maladaptation with the academic environment, the incidence of depressive symptoms, isolating feeling, and stress symptoms during the first year of the course as well as less academic performance in subsequent years [37]. In another study, Camp (2016) show that the academic optimism is positively correlated with surviving till finals and become well-qualified in the field, motivation, and academic achievement [39]. The second factor is communication. Effective communication is an essential element for nursing students to feel well-being and subsequently increases the level of their self-confidence, and motivation as well as their self-esteem [40]. Also Sheu et al. report that the inability to communicate with nurses is one of the most important stressors among senior

students[41], additionally academic competency is evaluated based on academic achievement, classmate relationship, and social behavior[42]. Furthermore, Marañón (2015) states that the student-instructor relationship is a determining factor in learning, as far as it makes progress in the learning process and leading to shaping students' identities as forthcoming nursing professionals [43] as well as increasing in their motivation for learning [44]. The third factor extracted is self-esteem/evaluation which is known as a prime predictor of stress management. In other words, Self-esteem indicates the degree of belief in the ability, importance, success, and individual’s competency. Having a high-level of self-esteem in managing the needs of nursing students during the practice course, and establishing a strong and therapeutic relationship with the patient is crucial [45]. In the present study, students also attempt to achieve self-esteem through knowledge enhancement, making trust, and support. The fourth extracted factor is self-awareness.This psychological component implies awareness of feelings, motivations, self-concept, and personality [39]. Since, self-awareness is a key for preventing unhealthy reaction to stress, therefore nursing has accepted Maslow's theory of motivation, and Rogers's view of self-awareness as fundamental issues of professional nursing [46], and it is mentioned as an antecedent to high-quality nursing care [47], so that awareness of identity as a nurse allows us to be aware of the positive and negative effects of the role. Also, the “nursing process” is often described as a problem-solving method, the nurse's self-awareness will help to answer this question, what is the problem?. On the other side, by understanding the effects of characteristics such as age, gender, and race on another person, the nurse can better gain the patient's trust and cooperation to stimulate patients' active participation [48]. The fifth factor extracted is trustworthiness. The development of resilience in nursing students hinges on a trust-based educational culture which paves the way for caring so nursing students in this culture can focus

on the needs of the patient [49]. In a systematic review of qualitative studies, Rørtveit et al. (2015) stated that nurses who facilitate the trust relationship engage, listen, and act as a patient supporter comprehensively [50]. In this study the whole students try to meet the patient's needs by establishing a trust-based relationship through strengthening nursing knowledge and for achieving this goal, they benefit from the support of family and friends. The last factor extracted is self-regulation. One of the protective factors in resilience is the development of self-regulation (emotional and behavioral regulation) [42], indeed Students who personally adjust their learning process will learn more effectively [51]. As far as Self-regulation is a deed that includes determining learning goals, adjusting personal effort, participating in time management, supervision, and evaluating existing performance [52] consequently, one must be directed to a specific goal or organization to motivate [53]. Achievement in theoretical courses, and becoming an expert in the clinical settings are considered as the goals of nursing students [54], thus students try to maintain and promote resilience by determining and achieving reasonable and task-based goals. In this regard, according to the practical identity of nursing, the more the students do clinical procedures, the more expert they become.

The CFA results detect the fitness of the final model of the NSARI in nursing students. Other construct validity indices also show the convergent and divergent validity in the final model. Convergent validity exists when the factors of the instrument are close together and explain a large variance. In other words, there is divergent validity when the extracted factors are separate from each other [20].

Limitations

Despite the validity and reliability of the inventory and final approval of the factor structure, the present study has some limitations that include the method of data gathering through the self-

report method, which can cause errors in reporting. Also, the design of the study is cross-sectional and data collected at one time, therefore to study the effects of time, and individuals' characteristics follow-up studies will be valuable to investigate the changes in the resilience factor structure. In addition, the percentage of extractive variance is almost less than 50%, which is accepted by many psychometric studies. To our knowledge, the NSARI is the first inventory to measure resilience in a theoretically driven way that has the potential to track and predict resilience in nursing students because of evidence of validity and reliability in this population. Further studies are recommended to investigate the conceptual structure of this inventory to gather more evidence regarding the psychometric properties of the inventory. Moreover, it seems necessary for further studies in nursing students in other cultures to confirm the findings of the present study.

Acknowledgements

We appreciate from nursing students who participated in this study, and also all the tutor and nurses who helped to carry out this study.

References

1. Pourafzal F, Seyedfatemi N, Inanloo M, Haghani H. Relationship between Perceived Stress with Resilience among Undergraduate Nursing Students. Hayat. 2013;19(1):1-12(Persian).
2. Porter SL. First year nursing students' perceptions of stress and resilience during their initial clinical placement and the introduction of a stress management app: a mixed methods approach: Edinburgh Napier University; 2019.
3. Stephens TM. Nursing student resilience: a concept clarification. Nursing forum. 2013;48(2):125-33.
4. Hwang E, Shin S. Characteristics of nursing students with high levels of academic resilience: A cross-sectional study. Nurse education today. 2018;71:54-9.
5. Reyes AT, Andrusyszyn MA, Iwasiw C, Forchuk C, Babenko-Mould Y. Resilience in nursing education: an integrative review. Journal of Nursing Education. 2015;54(8):438-44.
6. Mealer M, Schmiege SJ, Meek P. The Connor-Davidson Resilience Scale in critical care nurses: a psychometric analysis. Journal of nursing measurement. 2016;24(1):28.
7. Shean M. Current theories relating to resilience and young people. Victorian Health Promotion Foundation: Melbourne, Australia [Internet]. 2015. Available from: http://evidenceforlearning.org.au/assets/Grant-Round-II-Resilience/Current-theories-relating-to-resilience-and-young-people.pdf.
8. Windle G, Bennett KM, Noyes J. A methodological review of resilience measurement scales. Health and quality of life outcomes. 2011;9(1):8.
9. van der Meer CAI, te Brake H, van der Aa N, Dashtgard P, Bakker A, Olff M. Assessing Psychological Resilience: Development and Psychometric Properties of the English and Dutch Version of the Resilience Evaluation Scale (RES). Frontiers in Psychiatry. 2018;9(169). doi: 10.3389/fpsyt.2018.00169.
10. Chmitorz A, Kunzler A, Helmreich I, Tüscher O, Kalisch R, Kubiak T, et al. Intervention studies to foster resilience – A systematic review and proposal for a resilience framework in future intervention studies. Clinical Psychology Review. 2018;59:78-100. doi: https://doi.org/10.1016/j.cpr.2017.11.002.
11. Lock S, Rees CS, Heritage B. Development and validation of a brief measure of psychological resilience: The state–trait assessment of resilience scale. Australian Psychologist. 2020;55(1):10-25.
12. Karaırmak Ö. Establishing the psychometric qualities of the Connor–Davidson Resilience Scale (CD-RISC) using exploratory and confirmatory factor analysis in a trauma survivor sample. Psychiatry Research. 2010;179(3):350-6. doi: https://doi.org/10.1016/j.psychres.2009.09.012.
13. Windle G. What is resilience? A review and concept analysis. Reviews in Clinical Gerontology. 2011;21(2):152-69.
14. Elo S, Kyngäs H. The qualitative content analysis process. Journal of advanced nursing. 2008;62(1):107-15. doi: https://doi.org/10.1111/j.1365-2648.2007.04569.x.
15. Polit DF, Yang F. Measurement and the measurement of change: a primer for the health professions: Wolters Kluwer Health; 2015.
16. Lawshe CH. A quantitative approach to content validity. Personnel psychology. 1975;28(4):563-75.
17. Polit DF, Beck CT, Owen SV. Is the CVI an acceptable indicator of content validity? Appraisal and recommendations. Research in nursing & health. 2007;30(4):459-67.
18. Cristobal E, Flavian C, Guinaliu M. Perceived e- service quality (PeSQ). Managing service quality: An international journal. 2007.
19. Pahlevan Sharif S, Sharif Nia H. Structural equation modeling with AMOS. Tehran: Artin Teb. 2018.
20. Ali-Abadi T, Talepasand S, Boyle C, Sharif Nia H. Psychometric properties of The Baruth Protective Factors Inventory among nursing students. PloS one. 2020;15(6):e0233760.
21. Fok D. Development and testing of a Low Vision Product Selection Instrument (LV-PSI): A mixed-methods approach. 2011.
22. Munro BH. Statistical methods for health care research: lippincott williams & wilkins; 2005.
23. Hahs-Vaughn DL. Applied multivariate statistical concepts: Routledge; 2016.
24. Gatignon H. Statistical analysis of management data: Springer; 2003.
25. Fornell C, Larcker DF. Evaluating structural equation models with unobservable variables and measurement error. Journal of marketing research. 1981;18(1):39-50.
26. Javali SB, Gudaganavar NV, Raj SM. Effect of varying sample size in estimation of coefficients of internal consistency. 2011.

27. Moss S, Prosser H, Costello H, Simpson N, Patel P, Rowe S, et al. Reliability and validity of the PAS-ADD Checklist for detecting psychiatric disorders in adults with intellectual disability. Journal of intellectual disability research. 1998;42(2):173-83.
28. Nunnally JC, Bernstein I. Psychometric theory. New York: McGraw-Hill; 1994.
29. Sharif Nia H, Goudarzian AH, Shahrokhi A, Boyle C, Wilson MS, Hosseinigolafshani SZ. Psychometric properties of End- of- Life Caregiving Experience Appraisal Scale: Iranian critical care nurses. Nursing in Critical Care. 2019.
30. Hair J, Black W, Babin B, Anderson R. Multivariate data analysis William C. Black 7th ed Harlow: Pearson Education Limited. 2014.
31. Huang S-L, Hsieh C-L, Wu R-M, Tai C-H, Lin C-H, Lu W-S. Minimal detectable change of the timed “up & go” test and the dynamic gait index in people with Parkinson disease. Physical therapy. 2011;91(1):114-21.
32. Mokkink LB, Terwee CB, Patrick DL, Alonso J, Stratford PW, Knol DL, et al. The COSMIN checklist for assessing the methodological quality of studies on measurement properties of health status measurement instruments: an international Delphi study. Quality of life research. 2010;19(4):539-49.
33. Yekaninejad M, Mohammadi-Zeidi I, Akaberi A, Golshan A, Pakpour A. Validity and reliability of the kidney disease quality of life-short form (KDQOL-SFTM 1.3) in iranian patients. 2012.
34. Creswell JW. Research design: qualitative, quantitative, and mixed methods approaches. . 4th ed ed. Thousand Oaks, California: SAGE Publications.; 2014.
35. Southwick SM, Bonanno GA, Masten AS, Panter-Brick C, Yehuda R. Resilience definitions, theory, and challenges: Interdisciplinary perspectives. European journal of psychotraumatology. 2014;5(1):253-38.
36. Losoi H, Turunen S, Wäljas M, Helminen M, Öhman J, Julkunen J, et al. Psychometric properties of the finnish version of the resilience scale and its short version. 2013.
37. Soares MH, Luís MAV, Corradi-Webster CM, Martins JT, Hirata AGP. Psychological concept of optimism and drug use among nursing students. Acta Paul Enferm. 2011;24(3):393-9.
38. Singh C, Sharma S, Sharma RK. Level of stress and coping strategies used by nursing interns. Nursing and Midwifery Research Journal. 2011;7(4).
39. Camp NE. The relationship of a course grade to hope and optimism in nursing students' academic success: University of Alabama Libraries; 2016.
40. Subke J, Downing C, Kearns I. Practices of caring for nursing students: A clinical learning environment. International Journal of Nursing Sciences. 2020.
41. Sheu S, Lin H-S, Hwang S-L. Perceived stress and physio-psycho-social status of nursing students during their initial period of clinical practice: the effect of coping behaviors. International journal of nursing studies. 2002;39(2):165-75.
42. Masten AS, Powell L. A Resilience Framework for Research, Policy. Resilience and vulnerability: Adaptation in the context of childhood adversities. 2003:1.
43. Marañón AA, Pera MPI. Theory and practice in the construction of professional identity in nursing students: A qualitative study. Nurse education today. 2015;35(7):859-63.
44. Jamshidi N, Molazem Z, Sharif F, Torabizadeh C, Najafi Kalyani M. The challenges of nursing students in the clinical learning environment: A qualitative study. The Scientific World Journal. 2016;2016. doi: https://doi.org/10.1155/2016/1846178.
45. Edwards D, Burnard P, Bennett K, Hebden U. A longitudinal study of stress and self-esteem in student nurses. Nurse education today. 2010;30(1):78-84.
46. Eckroth-Bucher M. Self-awareness: A review and analysis of a basic nursing concept. Advances in Nursing Science. 2010;33(4):297-309.
47. Kirkpatrick AJ, Cantrell MA, Smeltzer SC. Relationships among nursing student palliative care knowledge, experience, self-awareness, and performance: An end-of-life simulation study. Nurse education today. 2019;73:23-30.
48. Rawlinson JW. Self awareness: conceptual influences, contribution to nursing, and approaches to attainment. Nurse education today. 1990;10(2):111-7.
49. Amsrud KE, Lyberg A, Severinsson E. Development of resilience in nursing students: A systematic qualitative review and thematic synthesis. Nurse education in practice. 2019;41:102621.
50. Rørtveit K, Hansen BS, Leiknes I, Joa I, Testad I, Severinsson E. Patients' experiences of trust in the patient-nurse relationship-a systematic review of qualitative studies. 2015.
51. Jouhari Z, Haghani F, Changiz T. Factors affecting self-regulated learning in medical students: a qualitative study. Medical education online. 2015;20(1):28694.

52. Phan HP. Critical thinking as a self-regulatory process component in teaching and learning. Psicothema. 2010;22(2):284-92.
53. Raman J. Nursing student success in an associate degree program. Teaching and Learning in Nursing. 2013;8(2):50-8.
54. Cho M-H, Shen D. Self-regulation in online learning. Distance education. 2013;34(3):290-301.

Figure1 Click here to access/download;Figure;fig.tif

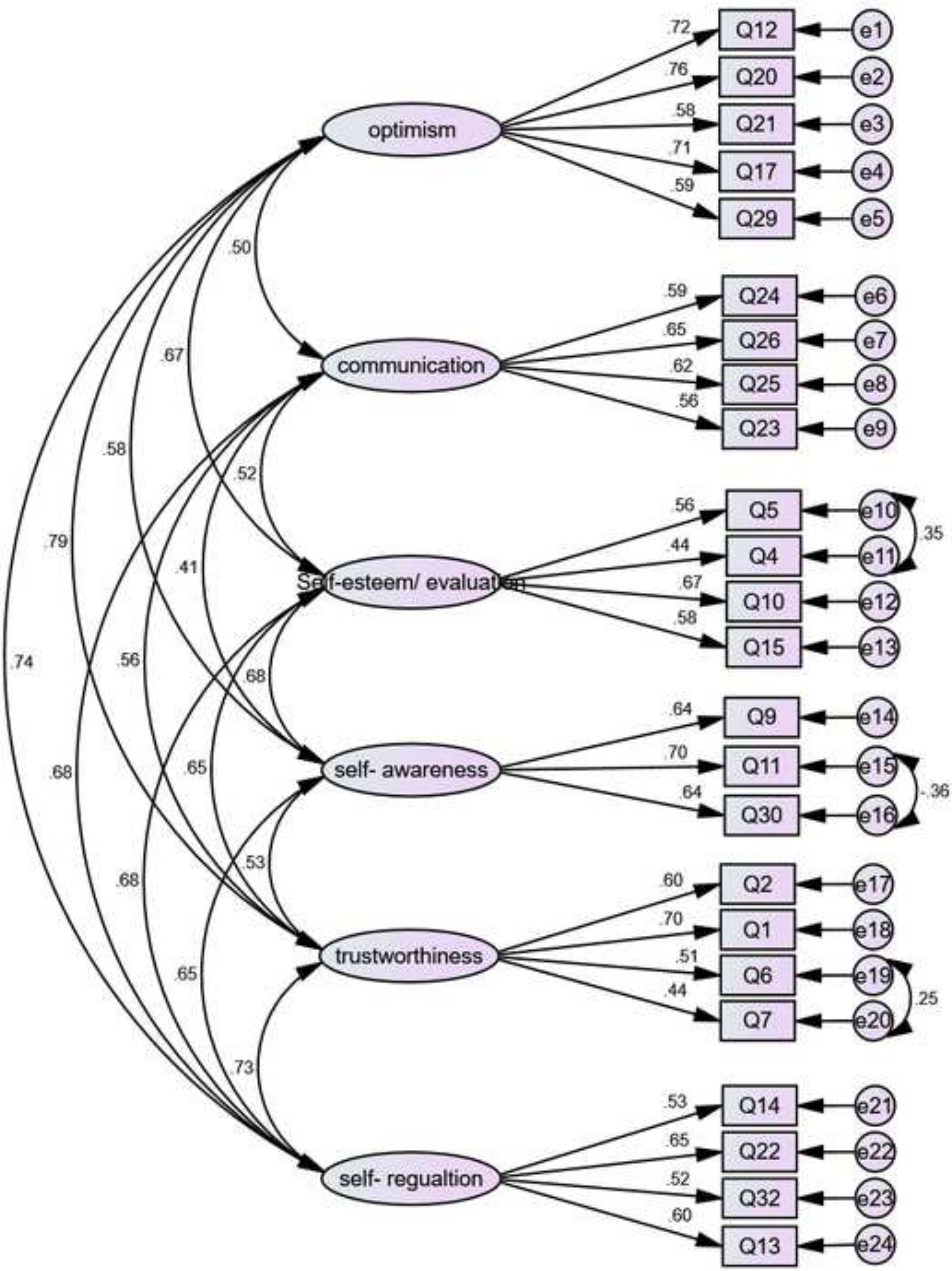

Figure2

Click here to access/download;Figure;fig2.tif

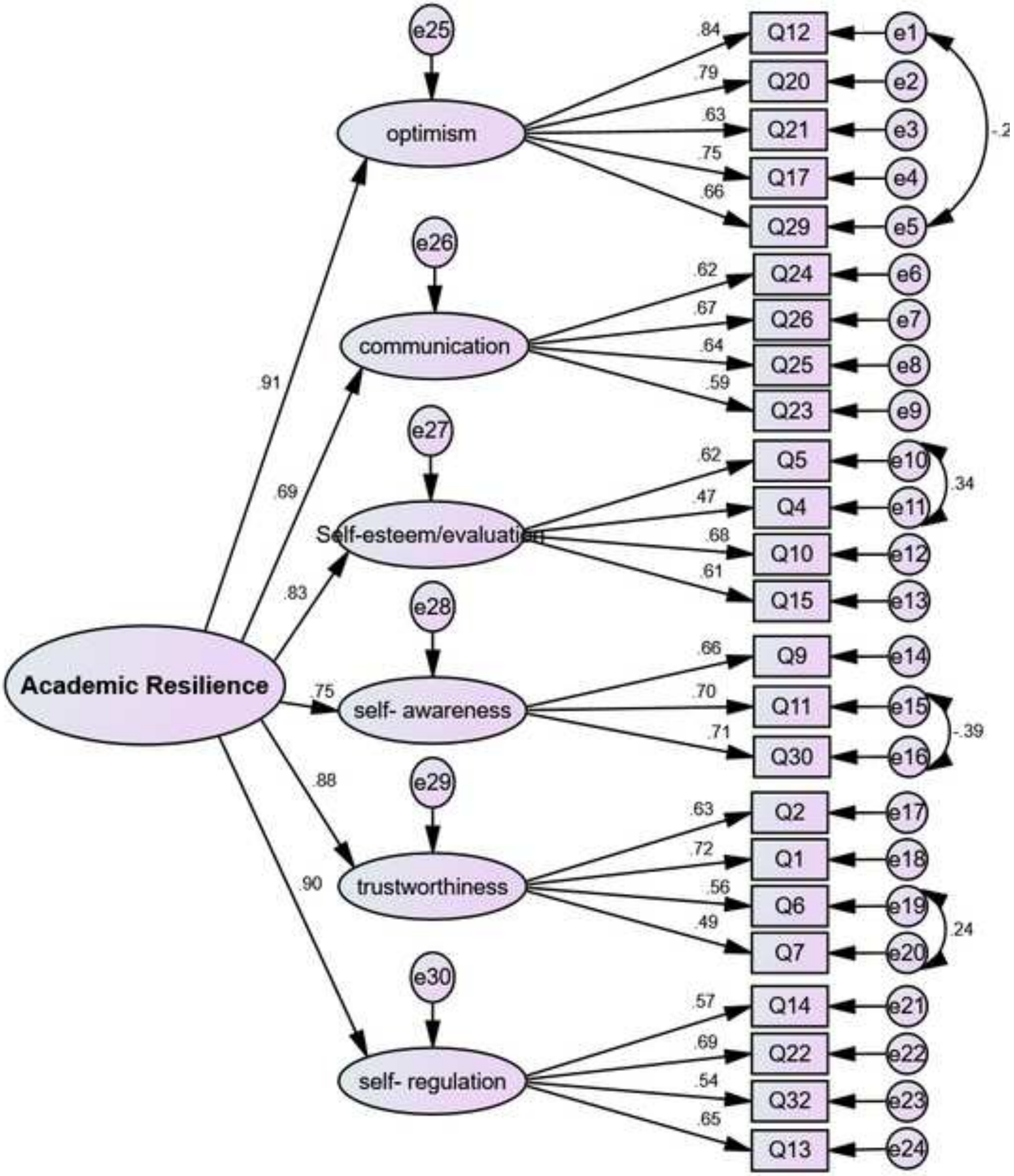

Click here to access/download
Supporting Information
academic resilience405.sav
