# Supplementary material for: Development and psychometric properties of the Nursing Student Academic Resilience Inventory (NSARI): A mixed-method study
Source: PLoS One. 2021 Jun 15;16(6):e0252473. doi: 10.1371/journal.pone.0252473 (PMC8205183; doi:10.1371/journal.pone.0252473)
Supplement: Attachment — Submitted filename: Response to reviewers.doc [file pone.0252473.s003.doc]

**Dear Editor-in-Chief of PLOS ONE Journal**

**Dr. Joerg Heber**

Response to reviewers

Thank you for your thorough review and consideration of our manuscript “Development and psychometric properties of the **Nursing Student Academic resilience** Inventory (NSARI): A Mixed-Method study" that was submitted to PLOS ONE Journal.

We believe that the comments and suggestions that were recommended by the reviewers have informed a much improved and more fully developed paper that will offer an important contribution to the field.

Given the word length requirements for clinical research briefs, we are somewhat stymied by the extent of details we can provide, However, and of critical importance, attention has been paid to developing a clearer rationale that addresses the significance of this work. More information has been provided to describe the measures used and data analysis conducted and this revised paper has more clarity about the comments and suggestions the reviewers has proposed.

We have highlighted the changes that were undertaken in response to your comments in the revised manuscript. A response to each of your comments is below.

Please feel free to contact me with any questions and concerns. I look forward to hearing from you regarding the manuscript.

Thanks for your kind attention to the manuscript.

Sincerely, Corresponding author

**Reviewer Comments to the Author**

| **Reviewer 1** | **Response** |
| --- | --- |
| 1. It is better to write Sample size same in the abstract (15) and the main study (13). | Due to this study is a mixed method study and has qualitative and quantitative phase , also in quantitative phase has several stage so the number of participants is different in every stage but overall, the number of participants in the qualitative stage was 15 participant and in final stage of psychometric (EFA, CFA) was 405.    P: 2 Methods: individual interviews are conducted with 15 participants  psychometric analysis …….. with a sample size of 405 nursing students. |
| 2. If you write the Abbreviations (minimum) in the study readers can read easily your article. | P:7,8,9,13  The abrevation are written, and highlited. |
| 3. You write the conclusion section in the abstract. It is better to write in the main study although it is optional (journal requirement) | The abstract was written based on instruction for author. |
| 4.Exploratory factor of the NSARI seems very good. | Thank you for your kind comment. |
| 5. in Table 2 better to write abbreviations below the table. | P: 12.  It is written , and highlighted |

| Reviewer2 | Response |
| --- | --- |
| Grammar, word choice & sentence structure issues are barriers to fully comprehending the manuscript content. Most of the manuscript is also written in present/future tense; if the studies are completed, it should all be written in past tense. | The present/future tense has been changed to past tense. |
| There is not enough detail about current instruments and what makes this instrument necessary. | P: 4  Diverse approaches to measuring resilience affected by different nature of potential risk factors and protective processes have led to contradictions . While establishing the resilience concept as a meaningful concept in research and clinical practice, it is inevitable to determine its distinctive factors and measure its factors in a valid and reliable method . Different studies have found different factor structures of resilience tools in different populations and cultures . Even though measuring resilience has done by a number of researchers such as Charney, Wagnild, Kobasa, but researchers and clinicians do not have strong evidences about the choice of resilience measurement tools and may make inappropriate choices in the field of study . Notwithstanding the prevalence of stress and the obligation to design an appropriate tool to assess students' resilience, it is not yet clear which factors measure the resilience in nursing students |
| P. 7 lines 150-151: the authors state “There is no missing data in the study due to completing the surveys by an online Survey software (Porsline).” The reviewer does not understand how having surveys completed online assures no missing data | In online Survey software responders have to respond all question because of following and responding one question was dependent on answering previous question, so much so,there was no missing data. |
| A description of the factors comes in the discussion section, but there is lacking a robust description of these in the results section | P:11  Items 12, 20, 21, 17,29 were assumed to mention optimism, items 24, 26,25,and 23 were reflected communication, Self-esteem/ evaluation was attributed to items 5,4,10,15, also items 9,11,30 composed Self-awareness, items 2,1,6,7 formed trustworthiness, and items 14,22,32, and 13 were hypothesized to reflect the Self-regulation (Table 2). |
| The reviewer believes that content in this manuscript was ambitious and likely too much to include in a single manuscript. Ultimately, this detracted significantly from fully comprehending and appreciating the processes of instrument development and the value of this newly developed instrument. | Due to avoiding salami publication, this article has been written as mixed method study.But we are somewhat stymied by the extent of details we can provide, However, and of critical importance, attention has been paid to developing a clearer rationale that addresses the significance of this work. |

| **Reviewer3** | **Response** |
| --- | --- |
| 1- kindly check the grammar and ensure correct sentence structures | It is done. |
| 2- You indicated using Stephen's Resilience Model. This is a good model but I don’t see from your writing how this model informed your research. | P: 3  In this study Stephen's resilience model was used which emphasized the importance of defining the concept of resilience as a process, also was proposed in nursing student setting.  P: 5  Exploratory questions were asked following the main questions that were derived from the Stephen resilience model. The main questions included “explain about the situation experienced stress during nursing education.”, “which protective factors did you apply in these situations?”, and “explain about cumulative success do you have acquired?”. |
| 3-The title and introduction look as if you are developing an entirely new tool. However, the factor structures seem to suggest that you were validating the NSARI tool in the Iranian context. Can you please clarify this? | P:5,6  The data in the qualitative phase were collected through semi-structured interviews based on resilience Stephen's model in nursing students. Item generation and developing inventory were done based on the categories extracted from the qualitative phase. Then, psychometric properties were evaluated in the quantitative phase. …….  the first draft of the inventory is developed based on the findings of the directed content analysis. |
| 4- The qualitative results did not indicate which items were generated and how they were elucidated from the qualitative study. It will be good to get a clear path on how items are generated from the interview data before psychometric analysis. At the moment, your qualitative results did not capture that. It is advisable to, at least, state the themes, categories, and sub-categories backed by participant quotes. This will provide evidence on how you arrived at the items that are used in the quantitative phase | P:10  An example of how the data were analyzed is shown in Table1.  P: 9  These codes were allocated to three themes, 9 categories (perceived stress: environment, relationship, Social-standing, Burnout, moral distress; protective factor: personal, social; cumulative success: Well-being, enhanced coping /adaptive abilities), and 31 subcategories based on interviews in the field of nursing student and Stephen’s model. |
| 5- In line 132, you indicated the inventory was revised by 13 nursing students in the qualitative face validity stage. How were these revisions done because your qualitative methodology only talk about how the items would be developed, and not about revision | The psychometric properties (in **quantitative phase**) includes face validity, content validity, construct validity,and reliability. Face validity is assessed both qualitatively and quantitatively.In the qualitative face validity, participants are asked to read aloud the items, explain what each item means, and determine the problematic or ambiguous words for revision.Then items are revised and rewritten according to their views. Quantitative face validity is performed to determine the importance of each item. |
| 6- Under the results in the quantitative phase, Line 209-2016 seems to be talking about method rather than results. Can you move this to the methods section? | This paragraph is related to the result of face and content validity. |
| 7- In line 209-210, you indicated that “In the psychometric evaluation phase, confusing items are revised and duplicate, redundant items are merged and essential items are added based on the results of the face and content validity” How were these revisions and merging done. Where from the ‘essential items”?  Face validity, as we know is usually done by experts. Who were the ‘experts’ in your study? Please kindly explain how this was done as it forms an essential aspect of instrument development | P: 6  Face validity refers only to the appearance of the instrument to the responders; that is, an instrument appears to measure what the test constructor claims it measures and also motivate participants to respond the survey. In terms of qualitative validity, participants were asked to read the items aloud, explain the meaning of each item, and identify problematic or ambiguous words for reconsideration Face validity is not validity in the true sense and refers only to the appearance of the instrument to the layperson; that is, if upon cursory inspection, an instrument appears to measure what  the test constructor claims it measures, it is said to have face validity. ) If an instrument has face validity, the layperson is more apt to be motivated to respond, thus its presence may serve as a factor in increasing response rate. Face validity,  when it is present, however, does not provide evidence for validity, that is, evidence that the instrument actually measures what it purports to measure). Carolyn Feher Waltz, Ora Lea Strickland, Elizabeth R. Lenz, Measurement in Nursing and Health Research. Fourth Edition( |
| 8- I am not too conversant with your measurement of convergent and divergent validity. However, I do know that the common, and probably the best, way of determining divergent validity for instance is to measure the focal construct (in this case resilience) and another closely related concept using the same instrument. Divergent validity would be established when the two measures are weakly correlated. This is to ensure that the tool is not measuring other constructs. I know this may not have been your intent but it is worth knowing | The criterion of Fornell-Larcker was used to estimate the convergent and divergent validity based on the average variance extracted (AVE), the maximum variance (MSV).  (Fornell C, Larcker DF. Evaluating structural equation models with unobservable variables and measurement error. Journal of marketing research. 1981;18(1):39-50.  Factor Analysis and Discriminant Validity: A Brief Review of Some Practical Issues ) |

| **Reviewer4** | **Response** |
| --- | --- |
| . Perhaps creating a specific section for the detailed description of the participants, another session for the details of the procedure, another session for the measurements and finally statistical analysis, could improve the reading considerably. | P:4  As far as this study is a mixed method study with two phase qualitative and quantitative with their own pariticipants and procedures , therefore the main devision is based on these phases. |
| For construct validity, the authors used a methodological approach that is commonly used in the field; however, the approach assumes a linear relationship between the items and the factor. Also, this methodology assumes a gaussian distribution of the outcome. This approach can be a problem for the construction of domains. Accurate methods for detecting relation between items are relevant for designing and determine the factor distribution. I suggest using Item Response Theory (IRT), specifically Graded Response Model (GRM; Samejima, 1969, 1997), Generalized Partial Credit Model (GPCM; Muraki, 1992) or multidimensional graded response model (MGRM). The IRT models examine the overall response patterns across all the items, while factor analytic methods examine covariances between the individual items. As a consequence of evaluating item response patterns, the parameter estimates provide insight into how the items work. Finally, the IRT estimates constructs assuming nonlinear relationship between latent traits and item responses. For IRT authors can use mirt package. It’s a powerful package and free tool for psychometric analyses: Chalmers, R. P. (2012). mirt: A multidimensional item response theory package for the R environment. Journal of Statistical Software, 48(6), 1-29. | Although ther authors believe that test development decisions could be improved by using additional information about item responses, the purpose of this study was development and psychometric properties of instrument, therefore item selection was based on indices of difficulty and discrimination (classic approach). (Introduction to Classical and Modern Test Theory. Linda Crocker, James Aigina)  P: 19 ; Also, in the suggestion section it is suggested:  To ensure that the instrument is psychometrically robust, its psychometric properties should be tested using different statistical methods and among different populations. Therefore, it is suggested that the Rasch model is used to test the NSARI psychometric properties of additional information. |
| Another suggested approach to construct validity is an exploratory structural equation modeling (see Asparouhov et al., 2009) approach. Asparouhov, Tihomir, and Bengt Muthén. "Exploratory structural equation modeling." Structural equation modeling: a multidisciplinary journal 16.3 (2009): 397-438. I’m agreed with authors, it’s necessary to estimate age and sex effect. However, I suggest reviewing the papers related to this issue of Rivera and Wim Van der Elst | The study was a mixed method study that represents an alternative methodological approach to traditional qualitative or quantitative research approaches to develope and psychometric properties of resilienceinstrument, the main purpose was not development of a resilience model, therefore item selection was based on indices of difficulty and discrimination (classic approach).  p:20  In addition the amount of data in the mixed method study is high, due to the word restriction in the article I would suggest in the suggestion section that it is consider in further study. |
| Please add the exclusion criteria that were used to enroll participants in the study | P:4  The study population includes all bachelor's nursing students who intended to participate in this study. The exclusion criteria was any circumstance that may interfere with study participation |
| Please provide information about how the sample size was calculated in each group | P: 5; In qualitative research there is not exact sample size and data saturation is importan.  To achieve maximum data diversity students were selected of both gender, different semesters, private and public universities. The interviews were perpetuated until data saturation.  For instruments with less than forty items, samples of 200 were reported to be adequate.( Lin SY, Tseng WT, Hsu MJ, Chiang HY, Tseng HC (2017) A psychometric evaluation of the Chinese version of the nursing home survey on patient safety culture. Journal of clinical nursing 26: 4664-4674.) |
| Provide more information about face and content validity data analyses technique | P:6  The psychometric properties (in quantitative phase) includes face validity, content validity, construct validity,and reliability. Face validity is assessed both qualitatively and quantitatively.In the qualitative face validity, participants are asked to read aloud the items, explain what each item means, and determine the problematic or ambiguous words for revision.Then items are revised and rewritten according to their views. Quantitative face validity is performed to determine the importance of each item.  *Face validity* is not validity in the true sense and refers only to the appearance of the instrument to the layperson; that is, if upon cursory inspection, an instrument appears to measure what the test constructor claims it measures, it is said  to have face validity. ) If an instrument has face validity, the layperson is more apt to be motivated to respond, thus its presence may serve as a factor in increasing response rate. Face validity, when it is present, however, does not provide  evidence for validity, that is, evidence that the instrument actually measures what it purports to measure). Carolyn Feher Waltz, Ora Lea Strickland, Elizabeth R. Lenz, Measurement in Nursing and Health Research. Fourth Edition(  p:7  Validity has then been commonly separated into various types—such as construct validity, content validity, onvergent and divergent validity, face validity, and factorial validity. COSMIN defi ned content validity as “the degree to which the content of an instrument is an adequate refl ection of the construct to be measured”. |
