# Supplementary material for: Development and psychometric properties of the Nursing Student Academic Resilience Inventory (NSARI): A mixed-method study
Source: PLoS One. 2021 Jun 15;16(6):e0252473. doi: 10.1371/journal.pone.0252473 (PMC8205183; doi:10.1371/journal.pone.0252473)
Supplement: Attachment — Submitted filename: Response to reviewers.doc [file pone.0252473.s004.doc]

**Dear Editor-in-Chief of PLOS ONE Journal**

**Dr. Joerg Heber**

Response to reviewers

Thank you for your thorough review and consideration of our manuscript “Development and psychometric properties of the **Nursing Student Academic resilience** Inventory (NSARI): A Mixed-Method study" that was submitted to PLOS ONE Journal.

We believe that the comments and suggestions that were recommended by the reviewers have informed a much improved and more fully developed paper that will offer an important contribution to the field.

Given the word length requirements for clinical research briefs, we are somewhat stymied by the extent of details we can provide, However, and of critical importance, attention has been paid to developing a clearer rationale that addresses the significance of this work. We have carefully reviewed the comments and have revised the manuscript accordingly. Our responses are given in a point-by-point manner below. Please find the revised manuscript that is edited according to reviewers’ and editors’ comments, and all these changes are highlighted.

We hope the revised version is now suitable for publication and look forward to hearing from you in due course.

Please feel free to contact me with any questions and concerns. I look forward to hearing from you regarding the manuscript.

Thanks for your kind attention to the manuscript.

**Sincerely, Corresponding author**

**Reviewer Comments to the Author**

| **Reviewer 1** | **Response** |
| --- | --- |
| 1. It is better to write Sample size same in the abstract (15) and the main study (13). | P: 2  Due to this study is a mixed method study and has qualitative and quantitative phase , also in quantitative phase has several stage so the number of participants is different in every stage but overall, the number of participants in the qualitative stage was 15 participant including 13 nursing students, one nurse and one trainer.  P:5  thirteen bachelor’s nursing students aged between 18 and 25 years old (5 women and 8 men), one female nurse, and one female trainer who had good communication skills and intended to participate in this study, were recruited using purposive sampling approach who have then participated only in the qualitative phase of the study. |
| 2. If you write the Abbreviations (minimum) in the study readers can read easily your article. | P:8,9,10  The abrevation are written, and highlited. |
| 3. You write the conclusion section in the abstract. It is better to write in the main study although it is optional (journal requirement) | The abstract was written based on instruction for author. |
| 4.Exploratory factor of the NSARI seems very good. | Thank you for your kind comment. |
| 5. in Table 2 better to write abbreviations below the table. | P: 12.  It is written , and highlighted |

| **Reviewer2** | **Response** |
| --- | --- |
| Grammar, word choice & sentence structure issues are barriers to fully comprehending the manuscript content. Most of the manuscript is also written in present/future tense; if the studies are completed, it should all be written in past tense. | The manuscript is edited by native English edit and the certificate letter is attached. |
| There is not enough detail about current instruments and what makes this instrument necessary. | P: 4  . However, different studies have found different factor structures of resilience tools in different populations and cultures . Accordingly, it may be due to the reason that despite the prevalence of stress and the obligation to design an appropriate tool to assess students' resilience, it is not yet clear which factors measure the resilience in nursing students. However, it was shown that having an understanding of resilient characteristics and the protective factors that promote resilience in nursing student can enable them to cope with adversity during their education period .  These diverse approaches to measure resilience affected by different natures of potential risk factors and protective processes have led to some contradictions . Due to establishing the resilience concept as a meaningful concept in research and clinical practice, it is inevitable to determine its distinctive factors and measure its factors using a valid and reliable method . Notably, the evaluation of designed interventions to promote resilience demands reliable and valid measures.  In this way, researchers and clinicians do not have strong evidences regarding the choice of resilience measurement tools, which may make inappropriate choices in the field of study . However, measuring resilience has been done by a number of researchers such as Charney, Wagnild, Kobasa. Additionally, the mean score of resilience in these studies varies from 83 to 104 because of using different generic scales. As a result, this creates difficulties in comparing mean score across studies, even if the nursing student experience similar adversities. In order to address these concerns, this study aimed to develop and design a new valid and reliable inventory for measuring resilience in nursing students. |
| P. 7 lines 150-151: the authors state “There is no missing data in the study due to completing the surveys by an online Survey software (Porsline).” The reviewer does not understand how having surveys completed online assures no missing data | P: 11  There was no missing data in this study due to completing the surveys by [Porsline. In this online survey software, responders must respond to all questions because following and responding one question depends on answering the previous one.](https://porsline.com/) |
| A description of the factors comes in the discussion section, but there is lacking a robust description of these in the results section | P:13,14  Correspondingly, the first factor was optimism, which can be defined as people's positive expectations of what is happening in their lives . In addition, having a positive optimistic attitude can help in more effectively dealing with stress . The second factor was labeled as communication defined as the exchange of information, ideas, and feelings among people who use speech or other means. Self-esteem/ evaluation as the third factor is known as a prime predictor of stress management indicateingthe degree of belief in the ability, importance, success, and individual’s competency . The fourth factor was identified as self-awareness. Accordingly, this psychological component implies awareness of feelings, motivations, self-concept, and personality . Trustworthiness as a worth demanding nurses to foster certain character attitudes and to strength effective task performance , was characterized as the fifth factor. Finally, self-regulation as the sixth factor was defined as the student’s attempt to manage learning processes focused on fulfilling goals . |
| The reviewer believes that content in this manuscript was ambitious and likely too much to include in a single manuscript. Ultimately, this detracted significantly from fully comprehending and appreciating the processes of instrument development and the value of this newly developed instrument. | P: 5  **Methods**  **Study design and Procedures**  This exploratory sequential mixed-method study was conducted in Iran from May 2019 to August 2020. It is assumed that all methods in this regard have bias and weaknesses, but based on triangulating data sources in this kind of design as well as the collection of both quantitative and qualitative data, the weaknesses of each form of data is neutralized. The data in the qualitative phase were collected through performing semi-structured interviews based on the resilience Stephen's model in nursing students. Item generation and developing inventory were also done based on the categories extracted from the qualitative phase. Finally, psychometric properties were evaluated in the quantitative phase.  **Qualitative phase:**  **Data collection**  **Data analysis**  **Quantitative phase:**  **Data collection**  **Data analysis** |

| **Reviewer3** | **Response** |
| --- | --- |
| 1- kindly check the grammar and ensure correct sentence structures | The manuscript is edited by native English edit and the certificate letter is attached. |
| 2- You indicated using Stephen's Resilience Model. This is a good model but I don’t see from your writing how this model informed your research. | P: 3,4  In this study, in line with different resilience theories, Stephen's resilience model of resilience in nursing students was used. Accordingly, this model emphasizes on the importance of defining the concept of resilience as a process performed based on antecedent/perceived adversities, attributes/protective factors, and consequences/cumulative successes. Moreover, the proposed model combines perceived adversities with the use of individual protective factors to cope and/or be adapted with the difficulties effectively. The cumulative successes of these events would lead to the increased resilience demonstrated by the enhanced coping/adaptive abilities and well-being status” .  P: 5,6  The data in the qualitative phase were collected through performing semi-structured interviews based on the resilience Stephen's model in nursing students.  Exploratory questions were asked following asking the main questions derived from the Stephen resilience model. The main questions included “explain about the situation experienced stress during nursing education.”, “which protective factors do you apply in these situations?”, and “explain about cumulative success that you have acquired”. |
| 3-The title and introduction look as if you are developing an entirely new tool. However, the factor structures seem to suggest that you were validating the NSARI tool in the Iranian context. Can you please clarify this? | P: 2  This study aimed to develop and validate a new inventory theory-driven labeled Nursing Student Academic Resilience Inventory.  P: 5  this study aimed to develop and design a new valid and reliable inventory for measuring resilience in nursing students.  P:5,6  The data in the qualitative phase were collected through performing semi-structured interviews based on the resilience Stephen's model in nursing students. Item generation and developing inventory were also done based on the categories extracted from the qualitative phase.  P:7  Data from the qualitative phase were used to develop Nursing Student Academic Resilience Inventory (NSARI) for the quantitative phase. |
| 4- The qualitative results did not indicate which items were generated and how they were elucidated from the qualitative study. It will be good to get a clear path on how items are generated from the interview data before psychometric analysis. At the moment, your qualitative results did not capture that. It is advisable to, at least, state the themes, categories, and sub-categories backed by participant quotes. This will provide evidence on how you arrived at the items that are used in the quantitative phase | P:12  An example of how the data were analyzed is shown in Table1.  P: 11,12  These codes were allocated to three themes, 9 categories (perceived stress: environment, relationship, Social-standing, Burnout, moral distress; protective factor: personal, social; cumulative success: Well-being, enhanced coping /adaptive abilities), and 31 subcategories based on interviews in the field of nursing student and Stephen’s model. |
| 5- In line 132, you indicated the inventory was revised by 13 nursing students in the qualitative face validity stage. How were these revisions done because your qualitative methodology only talk about how the items would be developed, and not about revision | P: 7  Face validity only refers to the appearance of the instrument to the responders; in other words, an instrument appears to measure what the test constructor intended to measure and also motivates the participants to respond to the survey. Confusing items were revised, duplicate, redundant items were merged, and essential items were added based on the results of the face and content validities. In terms of qualitative face validity, 13 nursing students were requested through convenience sampling to read the items loudly, explain the meaning of each item, and then identify problematic or ambiguous words for reconsideration. |
| 6- Under the results in the quantitative phase, Line 209-2016 seems to be talking about method rather than results. Can you move this to the methods section? | It is moved to the method section. |
| 7- In line 209-210, you indicated that “In the psychometric evaluation phase, confusing items are revised and duplicate, redundant items are merged and essential items are added based on the results of the face and content validity” How were these revisions and merging done. Where from the ‘essential items”?  Face validity, as we know is usually done by experts. Who were the ‘experts’ in your study? Please kindly explain how this was done as it forms an essential aspect of instrument development | P: 7  Face validity only refers to the appearance of the instrument to the responders; in other words, an instrument appears to measure what the test constructor intended to measure and also motivates the participants to respond to the survey. Confusing items were revised, duplicate, redundant items were merged, and essential items were added based on the results of the face and content validities. In terms of qualitative face validity, 13 nursing students were requested through convenience sampling to read the items loudly, explain the meaning of each item, and then identify problematic or ambiguous words for reconsideration.  Face validity is not validity in the true sense and refers only to the appearance of the instrument to the layperson; that is, if upon cursory inspection, an instrument appears to measure what the test constructor claims it measures, it is said to have face validity. ) If an instrument has face validity, the layperson is more apt to be motivated to respond, thus its presence may serve as a factor in increasing response rate. Face validity,when it is present, however, does not provide evidence for validity, that is, evidence that the instrument actually measures what it purports to measure). **Carolyn Feher Waltz, Ora Lea Strickland, Elizabeth R. Lenz, Measurement in Nursing and Health Research. Fourth Edition(** |
| 8- I am not too conversant with your measurement of convergent and divergent validity. However, I do know that the common, and probably the best, way of determining divergent validity for instance is to measure the focal construct (in this case resilience) and another closely related concept using the same instrument. Divergent validity would be established when the two measures are weakly correlated. This is to ensure that the tool is not measuring other constructs. I know this may not have been your intent but it is worth knowing | P:9  The criterion of Fornell-Larcker was used to estimate the convergent and divergent validity based on the average variance extracted (AVE), the maximum variance (MSV).  (**Fornell C, Larcker DF. Evaluating structural equation models with unobservable variables and measurement error. Journal of marketing research. 1981;18(1):39-50.).**  **Factor Analysis and Discriminant Validity: A Brief Review of Some Practical Issues** )  P: 9,16  Additionally, Heterotrait-monotrait ratio of correlations (HTMT) criterion was clculated. It was established that all values in the HTMT matrix table should be less than .85.  (Table 4). |

| **Reviewer4** | **Response** |
| --- | --- |
| . Perhaps creating a specific section for the detailed description of the participants, another session for the details of the procedure, another session for the measurements and finally statistical analysis, could improve the reading considerably. | P: 5  **Methods**  **Study design and Procedures**  This exploratory sequential mixed-method study was conducted in Iran from May 2019 to August 2020. It is assumed that all methods in this regard have bias and weaknesses, but based on triangulating data sources in this kind of design as well as the collection of both quantitative and qualitative data, the weaknesses of each form of data is neutralized. The data in the qualitative phase were collected through performing semi-structured interviews based on the resilience Stephen's model in nursing students. Item generation and developing inventory were also done based on the categories extracted from the qualitative phase. Finally, psychometric properties were evaluated in the quantitative phase.  **Qualitative phase:**  **Data collection**  **Data analysis**  **Quantitative phase:**  **Data collection**  **Data analysis** |
| For construct validity, the authors used a methodological approach that is commonly used in the field; however, the approach assumes a linear relationship between the items and the factor. Also, this methodology assumes a gaussian distribution of the outcome. This approach can be a problem for the construction of domains. Accurate methods for detecting relation between items are relevant for designing and determine the factor distribution. I suggest using Item Response Theory (IRT), specifically Graded Response Model (GRM; Samejima, 1969, 1997), Generalized Partial Credit Model (GPCM; Muraki, 1992) or multidimensional graded response model (MGRM). The IRT models examine the overall response patterns across all the items, while factor analytic methods examine covariances between the individual items. As a consequence of evaluating item response patterns, the parameter estimates provide insight into how the items work. Finally, the IRT estimates constructs assuming nonlinear relationship between latent traits and item responses. For IRT authors can use mirt package. It’s a powerful package and free tool for psychometric analyses: Chalmers, R. P. (2012). mirt: A multidimensional item response theory package for the R environment. Journal of Statistical Software, 48(6), 1-29. | Although ther authors believe that test development decisions could be improved by using additional information about item responses, the purpose of this study was development and psychometric properties of instrument, therefore item selection was based on indices of difficulty and discrimination (classic approach). (Introduction to Classical and Modern Test Theory. Linda Crocker, James Aigina)  P: 22,  To ensure that the instrument is psychometrically robust, its psychometric properties should be tested using different statistical methods such as the Rasch model and structural equation modeling, in order to estimate age and gender effects. |
| Another suggested approach to construct validity is an exploratory structural equation modeling (see Asparouhov et al., 2009) approach. Asparouhov, Tihomir, and Bengt Muthén. "Exploratory structural equation modeling." Structural equation modeling: a multidisciplinary journal 16.3 (2009): 397-438. I’m agreed with authors, it’s necessary to estimate age and sex effect. However, I suggest reviewing the papers related to this issue of Rivera and Wim Van der Elst | The study was a mixed method study that represents an alternative methodological approach to traditional qualitative or quantitative research approaches to develope and psychometric properties of resilienceinstrument, the main purpose was not development of a resilience model, therefore item selection was based on indices of difficulty and discrimination (classic approach).  P:22  it is suggested in limitation section. |
| Please add the exclusion criteria that were used to enroll participants in the study | P:5  The study population includes all bachelor's nursing students who intended to participate in this study. The exclusion criteria was any circumstance that may interfere with study participation |
| Please provide information about how the sample size was calculated in each group | Due to a mixed method study has a multiple phase and in different phase there is different numbers of participant so in every stage the number of participants and the way of selection is written.  P: 6  **Qualitative phase**  In qualitative research there is not exact sample size and data saturation is important.  To achieve maximum data diversity students were selected of both gender, different semesters, private and public universities. In qualitative studies, sample size cannot be pre-established, so much so the interviews were perpetuated until data saturation[**Morse, 2002 #1949**].  P: 7,8  **Quantitative phase**  **Data collection**  Data from the qualitative phase were used to develop Nursing Student Academic Resilience Inventory (NSARI) for the quantitative phase. Face validity was tested by 13 nursing students. Moreover, 11 experts in instrument development, nursing, and psychology were invited by email to validate this inventory in terms of content validity. All these experts were professors from the college of nursing and psychology and expertise in tool’s validation. Based on the Polit et al.’s study (2015), the size of the expert panel usually is between 3 and 12. For construct validity, those studies with the sample size of 100 or more were considered as “excellent”. Of note, the sample in CFA should be different from the sample used to finalize the instrument using EFA. According to the rule of thumb, a minimum of 200 cases in the analyses was considered to be enough. . Finally, 200 nursing students for EFA as well as 205 nursing students for CFA analysis were recruited in this study.  **Face and Content validity**  In terms of qualitative face validity, 13 nursing students through convenience sampling were requested to….  CVR and CVI Were done by 11 experts in instrument development, nursing, and psychology to determined  **Item Analysis**  36 nursing students were selected based on stratified sampling to recognize…  **Exploratory Factor Analysis**  in a cross‑sectional study, 200 nursing students of private and public universities from 6 different provinces in Iran participated in the study on account of convenience sampling.  **Confirmatory Factor Analysis**  Confirmatory factor analysis was done on a different sample including 205 nursing students who were selected by convenience sampling to…..  For instruments with less than forty items, samples of 200 were reported to be adequate.( **Lin SY, Tseng WT, Hsu MJ, Chiang HY, Tseng HC (2017) A psychometric evaluation of the Chinese version of the nursing home survey on patient safety culture. Journal of clinical nursing 26: 4664-4674.**)  P: 9  The Kaiser-Meyer-Olkin (KMO) statistic of sampling adequacy and the Bartlett test of sphericity were calculated to check the suitability of the data for factor analysis. |
| Provide more information about face and content validity data analyses technique | **P:7,8**  **Face and Content validity**  Face validity only refers to the appearance of the instrument to the responders; in other words, an instrument appears to measure what the test constructor intended to measure and also motivates the participants to respond to the survey. Confusing items were revised, duplicate, redundant items were merged, and essential items were added based on the results of the face and content validities. In terms of qualitative face validity, 13 nursing students were requested through convenience sampling to read the items loudly, explain the meaning of each item, and then identify problematic or ambiguous words for reconsideration. Following revising the items of the designed inventory according to the comments of the participants, at the qualitative face validity stage, quantitative face validity was assessed using the impact factor method based on the following equation…  Due to the COSMIN definition, content validity is “the degree to which the content of an instrument is an adequate reflection of the construct to be measured”. In this way, content validity ratio (CVR) was done by 11 experts to determine the essential items according to the cut-off point proposed in a study by Lawshe . |

**Additional Editor Comments**

| Introduction. Response to Reviewer #2-second point was not exhaustive | P:4  It is clarified. |
| --- | --- |
| The issue of missing data raised by Reviewer #2 is of maximum importance in research. . So, you should report your response statement into the manuscript when you described the methods. | P:11  It is written |
| As Reviewer #2 observed, a robust description of the factors that emerged is missing in the results section. | P: 13,14  It is explained |
| I would like to see a new paragraph named “Study design and Procedures” at the beginning of the Methods | P: 5  It is written. |
| Reviewer’s #3 point 2 should be thoroughly answered | P: 3,4  It is explained in more detailed. |
| To clarify any doubt like Reviewer’s #3 point 3, you should add the word “new. | P:2,5  It is written. |
| Reviewer‘s #3-point 6 request should be accomplished. Lines 229-230 describe the method of the quantitative phase; thus, it is better to move it above after line 135. | P: 7  It is moved |
| I ask you to eliminate the too generic Participants paragraph. Data collection of the qualitative phase should also include indication about recruitment of the participants and a more detailed description of the sample. | P:5,6  It is written |
| In the Quantitative phase a Data collection paragraph is totally missing | P;6,7 |
| The phrase “All the statistical analyses were calculated by SPSS-AMOS25 and SPSS R-Menu v2.0.” should be moved at the end of the quantitative phase, following line 204. | P: 10 |
| Ethical consideration: it is not enough to explain to the participants the volunteering and confidentiality of data, you should also explain the objectives of the study and collect their informed consent to participate in the study. | P:11  It is explained. |
| Results line 241: “Finally 13 items were eliminated from the analysis.” You should explain why, based on what criteria? | P:13  It explained. |
| Construct validity line 243: you should report the sample size of the sample used here and percentage of female, besides absolute number. Confirmatory factor analysis line 254. As in the previous paragraph, you should report here the sample size for this part, age and gender. | P:13  It is written. |
| ANOVA results line 290. Why did you not calculate multiple comparisons between subgroups based on semester? | P:17  It is done and reported. |
| Scoring items line 294: you should explain why you gave an indication for obtaining a total (summative) score instead of 6 factors scores, especially because in the Discussion you dedicated a large room to the description of the six constructs. | Composite measures often involve summing the item values to yield a total score, although sometimes, instrument developers recommend that the total score be the average across items so that the total score is on the same scale as the items.  (Measurement and the Measurement of Change. Polit (2015)) |
| Discussion: you cannot say “The CFA results detected the fitness of the final model”, because a number of fit indices you reported were under the recommended cutoff. Thus, you should underline and comment it. | P: 22  It is declared in limitation. |
| Limitations should be expanded: a probably sample disproportion regarding gender;  2) the use of validity indices based only on the internal structure of the scale. In future studies other methods could be used, like the heterotrait-monotrait (HTMT) ratio rates correlations, or the multitrait–multimethod matrix (MTMM), also correlating the instrument with external comparators (e.g, other measures of resilience);  3) the use of modification indices for fitting CFA results to your own data. This is an object of much disagreement among quantitative methodologists since this is a kind of “post hoc model modification” or a data-driven modification of the original hypothesized model. Other approaches could be used in future study to avoid this issue, like the exploratory structural equation modeling (ESEM) (see Asparouhov et al., 2009) as suggested by Reviewer #4. | P:22  the sample disproportion regarding gender; however, this is common because Nursing is a female-dominated profession  P: 9, 16  2--HTMT is calculated  3- p:9, 21  It is discussed in discussion section. |
| A linguistic revision is still needed; a few present tense is still there, and many phrases are not understandable. | The manuscript is edited by native English edit and the certificate letter is attached. |
