# Supplementary material for: Development and psychometric properties of the Nursing Student Academic Resilience Inventory (NSARI): A mixed-method study
Source: PLoS One. 2021 Jun 15;16(6):e0252473. doi: 10.1371/journal.pone.0252473 (PMC8205183; doi:10.1371/journal.pone.0252473)
Supplement: Attachment — Submitted filename: response to reviews.docx [file pone.0252473.s005.docx]

**Dear Academic Editor**

**Prof. Paola Gremigni**

Response to reviewers

Thank you for your thorough review and consideration of our manuscript “Development and psychometric properties of the **Nursing Student Academic resilience** Inventory (NSARI): A Mixed-Method study" that was submitted to PLOS ONE Journal.

We believe that the comments and suggestions that were recommended by the reviewers have informed a much improved and more fully developed paper that will offer an important contribution to the field.

We have carefully reviewed the comments and have revised the manuscript accordingly. Our responses are given in a point-by-point manner below. Please find the revised manuscript that is edited according to reviewers’ comments, and all these changes are highlighted, also the certification of Native English Edit company is uploaded.

We hope the revised version is now suitable for publication and look forward to hearing from you in due course.

Please feel free to contact me with any questions and concerns. I look forward to hearing from you regarding the manuscript.

Thanks for your kind attention to the manuscript.

**Sincerely, Corresponding author**

Reviewer #2

| What is lacking is a clear conceptualization of “resilience”. Describing the model components is not adequate as a definition of resilience. The reviewer believes this lack is at the core of the ambiguity found in the Introduction | P:3  Resilience evolved in the 1970s to understand why psychopathology was not always the consequences of risky environments in children [DeMichelis, 2016 #2004]. Resilience is described as a characteristic, process or outcome that depends on the theory accepted by the researcher[4]. Resilience’s theories can be outlined to three different models of resilience as “compensatory”, “protective”, or “inoculation/challenge” [DeMichelis, 2016 #2004] |
| --- | --- |
| The background and discussion appear to be much less clear and are in need of refinement | The background and discussion are refined  P: 3,4 (Introduction)  P: 19 (discussion)  The aim of present study was to develop a new inventory for measuring the resilience in nursing students. Although numerous tools have been designed in different contexts, there is only one study that was designed in nursing students. Yang et al study (2015) extracted 24 items through literature review and student interviews in two personal and contextual dimensions in Korean nursing. These dimensions included Self-confidence, Positivity, Coping ability, Emotional regulation, Structured style, Relationship, Social support (Yang YH, Kim EM, Yu M, Park S, Lee H. Development of the resilience scale for korean nursing college students. Korean Journal of Adult Nursing 2015; 27: 337-46.). As shown this study, resilience is multidimensional concept but some dimensions are the core of the resilience such as relationship and being able to cope. That was similar to the result of present study. |

Reviewer #6

| 1-the paper contains too much textbook statements with reiterations | below statements omitted and some statement paraphrases by Radan English Edit institute  P: 7  an instrument appears to measure and also motivates the participants to respond to the survey  P:8  Based on the Polit et al.’s study (2015), the size of the expert panel usually is between 3 and 12. For construct validity, those studies with the sample size of 100 or more were considered as “excellent”. Of note, the sample in CFA should be different from the sample used to finalize the instrument using EFA.  P:10  According to the COSMIN standards, besides validity and reliability, interpretability and scoring similarly are considered as important capabilities of a tool |
| --- | --- |
| 2- The paper needs a professional English editorial service. | The paper was edited again by www.NativeEnglishEdit.com  London  East End Road 27, N 3 3QT  United Kingdom |
| 3-Some statements are not supported with references as exampled, line 77-84.  Line 84 has improper quotation mark at the end. | P: 3  The paragraph mentioned to below reference that is cited: Stephens TM. Nursing student resilience: a concept clarification. Nursing forum. 2013;48(2):125-33.”  The quotation mark is deleted |
| 4- If the aim of the paper is based on the premise of line 86-87 (different studies have found different factor structures of resilience tools in different populations and cultures), this paper seems to serve only for Iranian nursing students. When there are several studies on resilience from various populations and cultures, the next step for study is to try to develop the integrated tool of resilience, rather than to add one more study of a specific population or culture. Or the paper should address the robust rationale on why the development of the resilience specifically for Iranian nursing student population is important in addition to those of previous study samples. Then the findings should compare resilience too variations among various populations and cultures. In fact, there have been many studies on resilience in nursing so far. | P: 3, 4  Previous studies conducted in Iran showed that nursing students are affected by various stressors… Resilience has been suggested as a beneficial solution for better dealing with challenges in nursing students.  The result of a review study yielded by Chmitorz et al. (2018) showed that various resilience scales have been developed [5]. Also, several studies have found **different factor structures** of resilience in different populations and cultures [6, 7]. In addition, there is **only one exploratory study in nursing student setting**[8]. Therefore, it is not yet clear which factors measure the resilience in nursing students [9] that seems to be related to different natures of potential risk factors and protective processes which have led to some contradictions while the evaluation of designed interventions to promote resilience demands reliable and valid measures. In this way, researchers and clinicians do not have strong evidences regarding the choice of resilience measurement tools, which may make inappropriate choices in the field of study [10].  P: 19  Although numerous tools have been designed in different contexts, there is only one study that was designed in nursing students. Yang et al study (2015) extracted 24 items through literature review and student interviews in two personal and contextual dimensions in Korean nursing. These dimensions included Self-confidence, Positivity, Coping ability, Emotional regulation, Structured style, Relationship, Social support [8]. As shown this study, resilience is multidimensional concept but some dimensions are the core of the resilience such as relationship and being able to cope. These results support the result of present study. Thomas et al (2016 ), also in integrative review declared factors that affect resilience in nursing students were grouped into three themes: support, time, and empowerment **.** |
| 5- line 94-96: Due to establishing the resilience concept as a meaningful concept in research and clinical practice, it is inevitable to determine its distinctive factors and measure its factors using a valid and reliable method [11]. It seems a little awkward sentence to me. The concepts of resilience have been established in many research and field. | It is deleted. |
| 6- The rationale for the study is not clear to me. On line 102-104: It is written, “Additionally, the mean score of resilience in these studies varies from 83 to 104[16, 17] because of using different generic scales. As a result, this creates difficulties in comparing mean score across studies, even if the nursing student experience similar adversities”. Then this study to develop and design a new valid and reliable inventory for measuring resilience in nursing students, seems to add one more different generic scale which may contribute to create difficulties in comparing mean across studies. | P:4  The result of a review study yielded by Chmitorz et al. (2018) showed that various resilience scales have been developed [6]. because resilience is a multidimensional construct that differes in different context, time, age, and life condition[Singh, 2010 #456]. several studies have found different factor structures of resilience in different populations and cultures [7, 8]. In addition, there is only one exploratory study in nursing student setting [Yang, 2015 #1793]. Therefore, it is not yet clear which factors measure the resilience in nursing students [9] that seems to be related to different natures of potential risk factors and protective processes which have led to some contradictions while the evaluation of designed interventions to promote resilience demands reliable and valid measures. In this way, researchers and clinicians do not have strong evidences regarding the choice of resilience measurement tools, which may make inappropriate choices in the field of study.  The paragraph about mean score is deleted. |
| 7-The criteria of subject seems still blurred. For example, on line 125, it is written ‘The exclusion criteria were any circumstance that may interfere with study participation.’ That is not the exclusion criteria. The circumstances of exclusion should be specified.  As well on line 145, it is written, ‘the samples were selected with maximum variation.’ What does it mean by maximum variation? | P:6  The exclusion criteria were any circumstance that may interfere with study participation such as speech and hearing impairment, having work experience in medical settings.  P:7  To assure data credibility, the samples were selected with maximum variation (private and public universities, different semesters, age, sex, and score). Also, to cover the variation of the phenomenon, a small data batch analyzed carefully first and then determined what additional data will be needed …. |
| 8- For ethical considerations, it is extremely important to protect the right of study participants and to maintain their security when they are students and the researchers are professors at same institutions. The circumstances to obtain the consent of participation from them need to be more addressed in terms of the relationship between participants and researchers, who proposed them to participate into the study, the condition of free refusal, no influence to any school activities by the study, etc. The description on line 254-259 is too simple.  As well the interview circumstances need to be explained more. The line 125 (interviews were conducted in a quiet room) is not enough  As well on line 145, it is written, ‘the samples were selected with maximum variation.’ What does it mean by maximum variation? | P: 11  Researchers were not professors at the same institutions. Therefore, there was any conflict of interest. The researcher referred to the education deputy and received the demographic list of students. In several sessions, students (in different educational semesters) invited to participate in the study. The aim of this study, data confidentiality, and voluntarily participation were explained.  P: 6  In the qualitative phase, the semi-structured individual interviews were conducted in a quiet room in the university or the educational class of hospital regarding to the student's choice. Students were selected through purposive sampling. The interviews started with asking an icebreaker question in which the individual is allowed to talk openly about the topic. Exploratory questions were asked following asking the main questions derived from the Stephen resilience model .  P:7  the samples were selected with maximum variation (private and public universities, different semesters, age, sex, and score). Also, to cover the variation of the phenomenon, a small data batch analyzed carefully first and then determined what additional data will be needed |
| 9- Discussion needs to be focused the comparison of resilience tool among subjects in relation to Stephen’s resilience model based on the premise and aim of the study | P: 16  Although numerous tools have been designed in different contexts, there is only one study that was designed in nursing students. Yang et al study (2015) extracted 24 items through literature review and student interviews in two personal and contextual dimensions in Korean nursing. These dimensions included Self-confidence, Positivity, Coping ability, Emotional regulation, Structured style, Relationship, Social support (Yang YH, Kim EM, Yu M, Park S, Lee H. Development of the resilience scale for korean nursing college students. Korean Journal of Adult Nursing 2015; 27: 337-46.). As shown this study, resilience is multidimensional concept but some dimensions are the core of the resilience such as relationship and being able to cope. That was similar to the result of present study |

Reviewer #7

| First, the author may want to develop a section called “Research Purpose and Specific Questions”. The authors only mentioned the purpose in one sentence on page 4. More detailed purpose can be addressed, and specific research questions can be developed. Specific research questions can guide the reader to better understand this study. | **p: 4**  **Research Purpose and Specific Questions**  To address the explained concerns, and regard to lack of specific resilience tool in nursing students the aim of present study in the first phase was to examine the stress, protective factors and cumulative success that students have acquired. The overall aim of the study was to design a new valid and reliable inventory for measuring resilience in nursing students. To achieve this aim, there were two research questions;  (1) Which factors are considered to be related to the resilience of nursing students?  (2) Which of these factors has statistically significant effect on the resilience of nursing students? |
| --- | --- |
| Second, the method section should be reorganized. As mentioned by a reviewer in the previous round, the participants section should provide more details regarding participants. Even though there are many phases with different participants, such as participants for interviews, EFA, and CFA. The same principle can be applied “Statistical Analyses”.  Further, there are many statistics mentioned in the method, but I could not find any relevant results, such as Mahalanobis d-squared. | It is reorganized overally.  P:5  **2.2 Participants**  In the qualitative phase, thirteen under graduated bachelor’s nursing students aged between 18 and 25 years old (5 female and 8 male), one female nurse, and one female trainer who had good communication skills and intended to participate in this study were recruited. Two students were dropout of keeping the education and one student was conditional student.  The quantitative phase included several stages. Face validity was tested by 13 nursing students (7female, 6 male) in different semesters. Moreover, 11 experts in instrument development, nursing, and psychology were invited by email to validate this inventory in terms of content validity. 200 nursing students for EFA as well as 205 nursing students for CFA analysis were recruited from 6 different provinces in Iran (age= 21.70 ±2.50; female=258, male= 147).  P:7  In the quantitative phase, the psychometric properties of the NSARI were assessed using face, content, and construct validities (exploratory and confirmatory factor analysis).  P:10  The presence of a multivariate outlier was assessed by Mahalanobis d-squared (*p*< .001),.  The Mahalanobis distance is a well-known criterion which may be used for detecting outliers in multivariate data.( Appropriate Critical Values When Testing for a Single Multivariate Outlier by Using the Mahalanobis Distance. Kay I. Penny. Journal of the Royal Statistical Society. Series C (Applied Statistics) [Vol. 45, No. 1 (1996)](https://www.jstor.org/stable/i349848), pp. 73-81 (9 pages) Published By: Wiley) |
| Third, in the results any statements should be along with supportive statistics. For instance, on page 11, the authors mentioned that “Finally, 13 items were eliminated from the analysis. Are there any statistics you should report to support your statistics? | P:8  Using a pilot study, the internal consistency was assessed before the construct validity assessment to recognize potential problems in the NSARI through estimating Cronbach’s alpha and inter‑item correlation. The items whose corrected item-total correlation score was less than 0.3, were then removed from the analysis [18].  P: 13  13 items were eliminated from the analysis due to their corrected item-total correlation scores was less than 0.3 in Item analysis. |

Reviewer #8:

| Line 111: which is triangulation to reduce Bias, there are several ways triangulation in several ways. For example, I see in the qualitative methods, there was author triangulation. | It is true that Validation of post positivist qualitative research relies upon using some strategies such as articulation of the research question., the use of more than one sources of data, providing a clear account of the process of data collection, *negative case and* triangulation of the findings*. In this study all of them have done*  *P:10.11*  2.5 Trustworthiness in the mixed-method study  To develop Self-reflexivity, after transcribing each one of the interviews, the researchers analyzed the obtained data to uncover their preunderstandings. As well, the researchers consulted with team members on the extracted codes and themes and then described the data analysis process in detail and finally provided clear citations in this regard.  To diminish the threats to the internal and external validities of the mix-method study, the participants with different experiences were selected. As well, none of the samples in the qualitative phase participated in the quantitative phase. Furthermore, designing the item pool was performed based on the main categories and sub-categories extracted in the qualitative phase. Consequently, all the stages of the study were carefully reviewed and verified by the other researchers [16, 36]. |
| --- | --- |
| Line 121:The author could try to maintain the language; use of man and woman vs male and female | It is revised |
| Line 122:The authors mention that for qualitative Data collection, they considered those with good communication skills- Why, Could this bias the study by excluding the those who had no good communication skills. What is good communication skills- was it at the discretion of the researcher to define good communication skills. Possibility that people with good communication are characteristically different from those with poor communication skills and probably have different resilient mechanisms. | Yes, it is true that people with good communication are characteristically different from those with poor communication skills and probably have different resilient mechanisms. Therefore, the researcher also interview with negative cases but the first interviewee was a female who was introduced by education deputy because she had good relationship with others and speak easily so we can reach a lot of data.  P:5  good communication skills including fluency in Persian language, no hearing or speech problems,  P: 5  Two students were dropout of keeping the education and one student was conditional student. |
| Line 123: The authors use the term intended to participate in the study. Not clear whether participants were consented to participate in the study. I like the fact the authors specify how the participants were selected. However wondering all people contacted participated in the study or there are some who could have decline and the possible reason if any decline | P: 11  The researcher referred to the education deputy and received the demographic list of students. In several sessions, students ( in different educational semesters) invited to participate in the study. The aim of this study, data confidentiality, and voluntarily participation were explained. In qualitative phase informed consent obtained was verbal due to in this way participants feel more intimate relationship for participating in interview sessions. the IRB at this site was inclined to this procedure.Thereafter, in the quantitative phase, surveys were completed through Porsline software.At the first of survey written informed consent was attached. The participants who did not want to participate were authorized not to complete the surveys. |
| Line 125: What us any circumstances-Could may be cite an example of how any circumstance that may interfere with study participation may look like | P: 6  The exclusion criteria were any circumstance that may interfere with study participation such as speech and hearing impairment, having work experience in medical settings. |
| Line 146:it is not clear to me If the researcher critically examined their own role, potential bias and influence during (a) formulation of the research questions (b) data collection, including sample recruitment and choice of location | P:10, 11  Trustworthiness in the mixed-method study  To develop Self-reflexivity , after transcribing the each interview, the researchers analyses the data to uncover their preunderstandings, also researchers consulted with team members about the extracted codes and themes and described data analysis process in detail and provided clear citations.  To diminish the threats to the internal and external validities of the mix-method study, the participants with different experiences were selected. As well, none of the samples in the qualitative phase participated in the quantitative phase. Furthermore, designing the item pool was performed based on the main categories and sub-categories extracted in the qualitative phase. Consequently, all the stages of the study were carefully reviewed and verified by the other researchers [15, 35]. |
| Line 256:Are you trying to describe verbal consent. other wise am worried that participants did not provide written informed consent. If writing, did it receive a waiver of consent from the REC? | P: 11  In qualitative phase informed consent obtained was verbal due to in this way participants feel more intimate relationship for participating in interview sessions. The IRB at this site was inclined to this procedure. Thereafter, in the quantitative phase, surveys were completed through Porsline software. At the beginning of inventory written informed consent was attached. |
| Line 259:This statement is not clear. if you had talked about the consent process here; this would not be necessary; Electronically, some authors will state that by clicking I agree to participate, is a taken as consent. But have to be given information about the study before accessing the survey | P:11  . At the beginning of the inventory written informed consent was attached and they declared their consent by clicking “I agree to participate”. |
