# Supplementary material for: Development and psychometric properties of the Nursing Student Academic Resilience Inventory (NSARI): A mixed-method study
Source: PLoS One. 2021 Jun 15;16(6):e0252473. doi: 10.1371/journal.pone.0252473 (PMC8205183; doi:10.1371/journal.pone.0252473)
Supplement: Attachment — Submitted filename: response to reviewer.docx [file pone.0252473.s006.docx]

**Dear Academic Editor**

**Prof. Paola Gremigni**

Response to reviewers

Thank you for your thorough and fast review and consideration of our manuscript “Development and psychometric properties of the **Nursing Student Academic resilience** Inventory (NSARI): A Mixed-Method study" that was submitted to PLOS ONE Journal.

We believe that the comments and suggestions that were recommended by academic editor have informed a much improved and more fully developed paper that will offer an important contribution to the field.

We have carefully reviewed the comments and have revised the manuscript accordingly. Our responses are given in a point-by-point manner below. Please find the revised manuscript that is edited according to comments, and all these changes are highlighted, also the article edited again by the Native English Edit company and the certification is uploaded.

We hope the revised version is now suitable for publication and look forward to hearing from you in due course.

Please feel free to contact me with any questions and concerns. I look forward to hearing from you regarding the manuscript.

Thanks for your kind attention to the manuscript.

**Sincerely, Corresponding author**

**Editor comments**

| ) Line 57 “Previous studies conducted in Iran showed that nursing students are affected by various stressors.” You should cite at least a few examples of such studies. | 1. Pourafzal F, Seyedfatemi N, Inanloo M, Haghani H. Relationship between Perceived Stress with Resilience among Undergraduate Nursing Students. Hayat. 2013;19(1):1-12(Persian).  2. Moaddeli Z, Ghazanfari Hesamabadi M. A survey on the students’ exam anxiety in the Fatemeh (PBUH) College of Nursing and Midwifery, Spring 2004. Strides in Development of Medical Education. 2005;1(2):65-72.  3. Nikanjam R, Barati M, Bashirian S, Babamiri M, Fattahi A, Soltanian A. Student-Life Stress Level and its Related Factors among Medical Students of Hamadan University of Medical Sciences in 2015. Journal of Education and Community Health. 2016;2(4):57-64. doi: <https://doi.org/10.21859/jech-02048>. |
| --- | --- |
| 2) Line 70 “In this study, Stephen's resilience model as protective model was 70 used.” You should cite a reference here where the reader can read about Stephen’s model. | P:3  In this study, Stephen's resilience model[4] |
| 3) Lines 202-204: the issue of the modification indices is a result already reported in the Results paragraph lines 319-320. | It is deleted from method section:  Regarding the modification indices, 4 pairs of measurement errors among the measured items of the first, third, fourth, and fifth factors were allowed to co-vary freely, which consequently improved the final model fit noticeably |
| 4) Please comment the under-threshold values of CFI and AGFI obtained in confirmatory factor analysis. | P:23  Moreover, a number of fit indices were found to be under the recommended cut-off point. To the best of our knowledge, the NSARI is the first inventory to measure resilience in a theoretically driven way that has the potential to track and predict resilience in nursing students, because of evidences of validity and reliability in this population. To ensure that the instrument is psychometrically robust, its psychometric properties should be tested using different statistical methods such as the Rasch model and structural equation modeling, in order to estimate age and gender effects. Further studies are recommended to investigate the conceptual structure of this inventory to gather more evidences regarding the psychometric properties of the inventory study |
| 5) When citing a reference, you should use the square parentheses method: line 79 Chmitorz et al. [5] not Chmitorz et al. (2018). See also citations at lines 365, 371, 386, 389, 397, and 418. | They are revised and highlighted. |
| 6) English editing is still needed: line 126 “regarding to”; lines 139-140; line 155; line 248; line 288 “participates”; line 370 “These results supported the result of the present study.” | The article is edited again by editors at NativeEnglishEdit.com and highlighted. |
